# Supplementary material for: A molecular descriptor of intramolecular noncovalent interaction for regulating optoelectronic properties of organic semiconductors
Source: Nat Commun. 2023 May 1;14:2500. doi: 10.1038/s41467-023-38078-4 (PMC10151346; doi:10.1038/s41467-023-38078-4)
Supplement: Supplementary file 1 — Supplementary Information [file 41467_2023_38078_MOESM1_ESM.pdf]

Supplementary Information

A molecular descriptor of intramolecular noncovalent interaction for regulating optoelectronic properties of organic semiconductors

Meihui Liu[‡1], *Xiao Han* [‡2], *Hao Chen* [2], *Qian Peng**[1], *Hui Huang**[2]

[1] School of Chemical Sciences, University of Chinese Academy of Sciences, Beijing, 100049, P. R. China.

[2] College of Materials Science and Opto-Electronic Technology & CAS Center for Excellence in Topological Quantum Computation & Key Laboratory of Vacuum Physics, University of Chinese Academy of Sciences, Beijing 100049, P. R. China

Table of Contents

1. Molecular structures (Supplementary Figure S1)

2. The Nature of Noncovalent Interactions (Supplementary Figure S2)

3. Calculated Conformational Energy Differences (Supplementary Table S1, Supplementary Figure S3)

4. Calculated Molecular Structure Parameter (Supplementary Table S2)

5. NBO analysis of NoCLs (Supplementary Figure S4)

6. The Establishment of the Descriptor (Supplementary Figure S5-S8)

7. Relaxation Energy (Supplementary Table S3, Supplementary Figure S9)

8. The Extension of the Descriptors (Supplementary Table S4-S5)

9. Supplementary Notes (Supplementary Figure S10)

10. Supplementary Methods (Supplementary Figure S11-S18)

11. The Experimental Characterizations (^{1}H-^{1}H NOESY, ^{77}Se and ^{125}Te NMR) of NoCLs (Supplementary Figure S19-S21)

12. UV–Vis Spectra and Stokes shifts (Supplementary Figure S22-S23, Supplementary Table S6)

13. NMR Spectra and Mass Spectrometry (Supplementary Figure S24-S51)

14. Supplementary references

1. Molecular structures

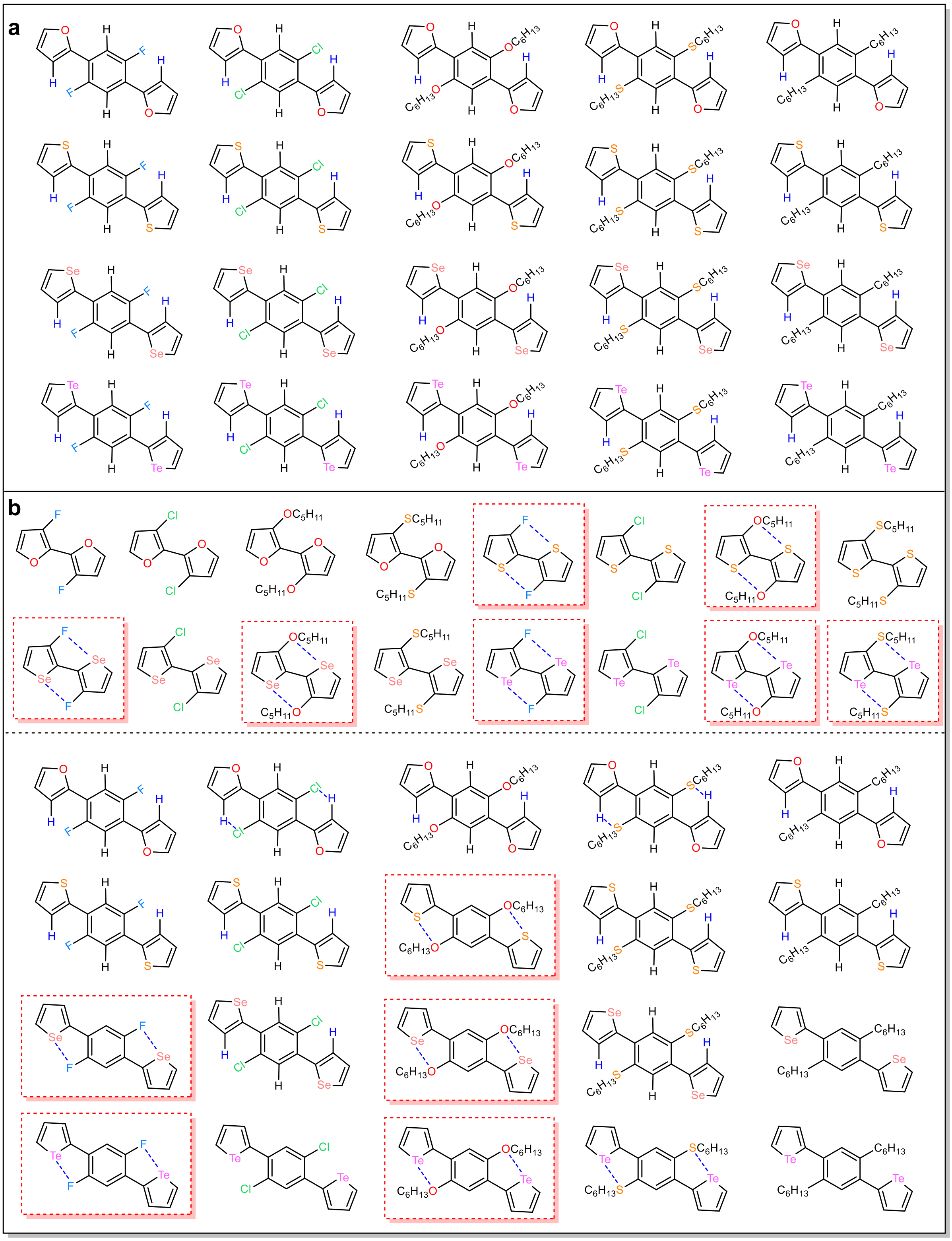

Supplementary Figure S1. Molecular structures of designed compounds in this work. a Type II: **PhM-H**(X···Y) in which θ is between 90° and 180° (20 molecules); **b** Molecular

structures for stable compounds. The red dotted line represents the presence of NoCLs based on *S*.

2. The nature of noncovalent interactions

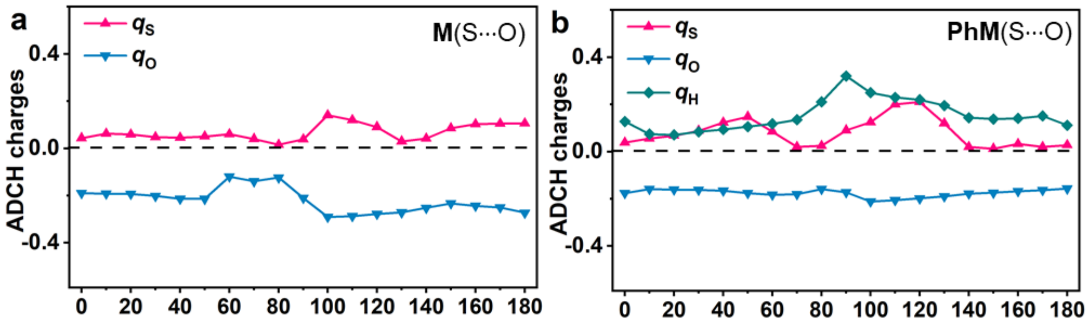

Supplementary Figure S2. The evolution of atomic dipole moment-corrected Hirshfeld (ADCH) charge as a function of the dihedral angle θ for a M(S···O) and b PhM(S···O). In **M**(S···O), the charges on S (q_S) and O (q_O) atoms are few and almost unchanged at θ∈[0°, 90°], which results in unchanged elecstatic interactions between them. In **PhM**(S···O), when θ changed from 0° to 180°, q_O remains almost a negative constant of ca. -0.18 a.u., q_H and q_S both are very small of <0.15 a.u. at θ∈[0°, 40°] and θ∈[140°, 180°] while they fluctuate at the range of 0.09~0.32 a.u. for q_H and 0.02~0.21 for q_S at θ∈[40°, 140°]. As a result, the electrostatic interactions are very weak with values less than -1.30 kcal mol^{-1} at θ∈[0°, 40°] and θ∈[140°, 180°] although they become a little stronger at θ∈[90°, 140°].

3. Calculated conformational energy differences

Supplementary Table S1. Calculated conformational energy differences of **PhM** and **PhM-H**.

$\Delta E = E_{\text{PhM}} - E_{\text{PhM-H}}$

Systems	E_{PhM} (kcal mol^{-1})	$E_{\text{PhM-H}}$ (kcal mol^{-1})	ΔE(kcal mol^{-1})
**O···F**	-557524.17	-557530.69	6.52
**O···Cl**	-1009778.39	-1009782.96	4.57
**O···O**	-823453.35	-823461.59	8.24
**O···S**	-1228797.56	-1228800.34	2.78
**O···C**	-729063.98	-729064.91	0.93
**S···F**	-962875.30	-962876.04	0.74
**S···Cl**	-1415128.73	-1415129.50	0.77
**S···O**	-1228807.15	-1228806.77	-0.39
**S···S**	-1634147.14	-1634148.86	1.72
**S···C**	-1134412.89	-1134412.92	0.03
**Se···F**	-474692.40	-474692.28	-0.12
**Se···Cl**	-926942.72	-926943.30	0.58
**Se···O**	-740620.29	-740618.54	-1.75
**Se···S**	-1145959.00	-1145959.00	0.00
**Se···C**	-646226.52	-646226.12	-0.40
**Te···F**	-473213.8	-473211.55	-2.25
**Te···Cl**	-925463.43	-925462.77	-0.66
**Te···O**	-739143.71	-739137.12	-6.59
**Te···S**	-1144481.86	-1144478.6	-3.26
**Te···C**	-644746.09	-644745.44	-0.65

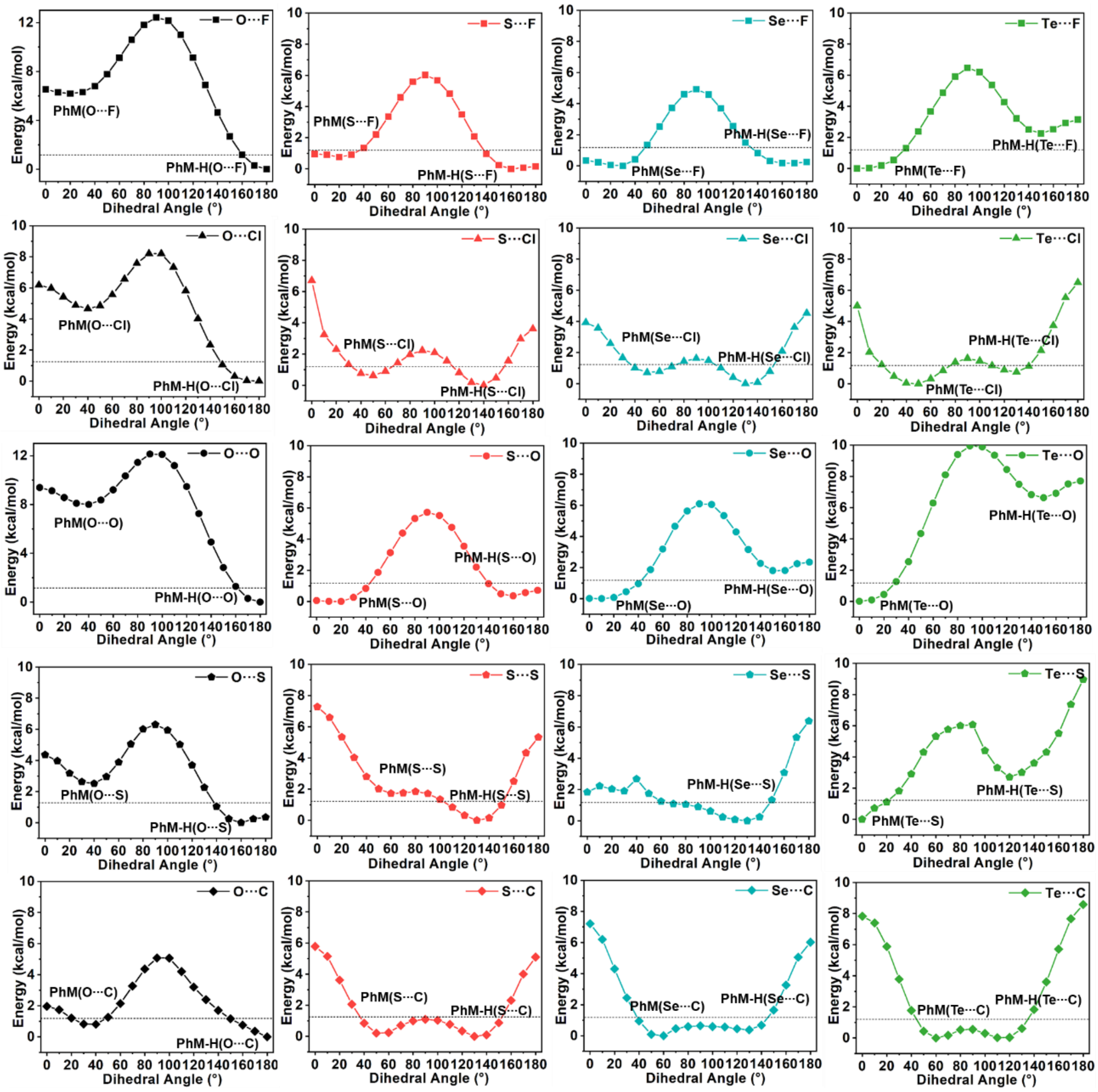

Supplementary Figure S3. Torsional (dihedral) potential energy surfaces (PES) derived at the B3LYP(D3)/6-31+G (d) level of type II. Note that 2 available thermal energy (2kT) at 298 K, approximately 1.2 kcal mol^{-1}, is provided as a reference (dashed line) because strength of ~2 kT are generally considered as the lower limit for conformational control, and any interaction weaker than that will likely be washed out by thermal fluctuations.

4. Calculated molecular structure parameter

Supplementary Table S2. The optimized dihedral angles (θ), orbital plane angles (α), bond lengths (*d*1 and *d*2) and descriptors (*S*) for 36 investigated systems. *d*v is the sum of van der Waals radii of two involved atoms.

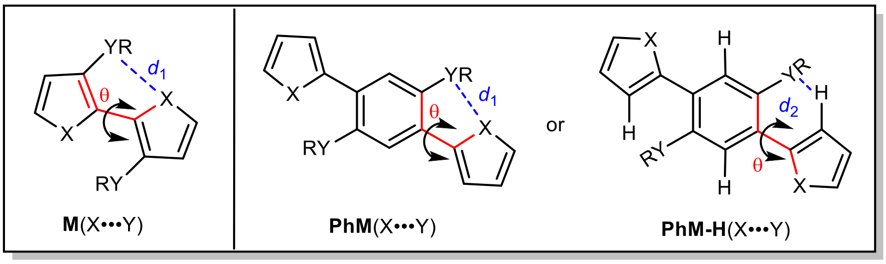

Systems	θ (°)	α (°)	*d*1 (Å)	*d*2 (Å)	*d*v (Å)	*S*
**M**(O···F)	0.00	168.74	3.022		2.99	0.001
**M**(O···Cl)	0.00	161.35	3.161		3.27	0.013
**M**(O···O)	0.07	167.78	2.948		3.04	0.009
**M**(O···S)	0.01	159.83	3.082		3.32	0.069
**M**(S···F)	0.01	166.44	2.950		3.27	0.138
**M**(S···Cl)	60.24	129.79	3.640		3.55	0.001
**M**(S···O)	0.01	165.11	2.854		3.32	0.348
**M**(S···S)	65.73	124.68	3.787		3.60	0.003
**M**(Se···F)	0.00	157.73	2.971		3.37	0.224
**M**(Se···Cl)	69.95	119.01	3.836		3.65	0.002
**M**(Se···O)	0.00	158.24	2.861		3.42	0.523
**M**(Se···S)	107.10	101.27	4.490		3.70	0.005
**M**(Te···F)	0.00	149.07	3.001		3.53	0.419
**M**(Te···Cl)	62.87	119.72	3.814		3.81	0.000
**M**(Te···O)	0.11	149.74	2.872		3.58	0.923
**M**(Te···S)	24.29	149.17	3.243		3.86	0.526
**PhM**(S···O)	17.29	161.77	2.748		3.32	0.511
**PhM**(Se···F)	17.25	154.49	2.870		3.37	0.346
**PhM**(Se···O)	1.47	158.85	2.750		3.42	0.849
**PhM**(Te···F)	0.04	150.19	2.890		3.53	0.697

**PhM**(Te···Cl)	46.21	134.38	3.487		3.81	0.049
**PhM**(Te···O)	0.04	150.65	2.801		3.58	1.217
**PhM**(Te···S)	48.75	133.05	3.430		3.86	0.086
**PhM**(Se···C)	65.12	124.04	3.569		3.6	0.000
**PhM**(Te···C)	65.14	119.96	3.713		3.76	0.000
**PhM-H**(O···F)	179.99	107.25		2.387	2.67	0.032
**PhM-H**(O···Cl)	164.65	110.04		2.672	2.95	0.034
**PhM-H**(O···O)	179.99	107.48		2.339	2.72	0.065
**PhM-H**(O···S)	161.08	109.00		2.620	3.00	0.062
**PhM-H**(S···F)	161.36	109.88		2.318	2.67	0.054
**PhM-H**(S···Cl)	139.19	101.09		2.852	2.95	0.001
**PhM-H**(S···S)	132.95	95.92		2.884	3.00	0.001
**PhM-H**(Se···Cl)	133.86	98.80		2.909	2.95	0.000
**PhM-H**(Se···S)	127.58	93.17		2.953	3.00	0.000
**PhM-H**(O···C)	161.47	98.89		2.723	2.72	0.026
**PhM-H**(S···C)	134.69	97.48		2.934	3.00	0.001

5. NBO analysis of NoCLs

$n(\mathrm{s})\rightarrow\sigma^*(\mathrm{sp}^2)$	$n(\mathrm{p})\rightarrow\sigma^*(\mathrm{sp}^2)$	$n(\mathrm{s})\rightarrow\sigma^*(\mathrm{sp}^2)$ and $n(\mathrm{p})\rightarrow\sigma^*(\mathrm{sp}^2)$	$n(\mathrm{sp}^2)\rightarrow\sigma^*(\mathrm{sp}^2)$
	**M**(O···F)<br>**M**(O···Cl)<br>**M**(S···S)<br>**M**(S···Cl)<br>**M**(Se···Cl)<br>**M**(Te···Cl)	**M**(S···F)<br>**M**(Se···F)<br>**M**(Te···F)<br>**M**(Te···S)	**M**(O···O)<br>**M**(O···S)<br>**M**(S···O)<br>**M**(Se···O)<br>**M**(Te···O)
**PhM**(Te···S)		**PhM**(Se···F)<br>**PhM**(Te···F)<br>**PhM**(Te···Cl)	**PhM**(S···O)<br>**PhM**(Se···O)<br>**PhM**(Te···O)
$n(\mathrm{s})\rightarrow\sigma^*(\mathrm{s})$		$n(\mathrm{s})\rightarrow\sigma^*(\mathrm{s})$ and $n(\mathrm{p})\rightarrow\sigma^*(\mathrm{s})$	$n(\mathrm{sp}^2)\rightarrow\sigma^*(\mathrm{s})$
**PhM-H**(O···S)<br>**PhM-H**(S···S)<br>**PhM-H**(S···C)<br>**PhM-H**(Se···S)		**PhM-H**(O···F)<br>**PhM-H**(O···Cl)<br>**PhM-H**(S···F)<br>**PhM-H**(S···Cl)<br>**PhM-H**(Se···Cl)	**PhM-H**(O···O)<br>**PhM-H**(O···C)
There are not orbital overlap at **M**(Se···S), **PhM**(Se···C), **PhM**(Te···C)			

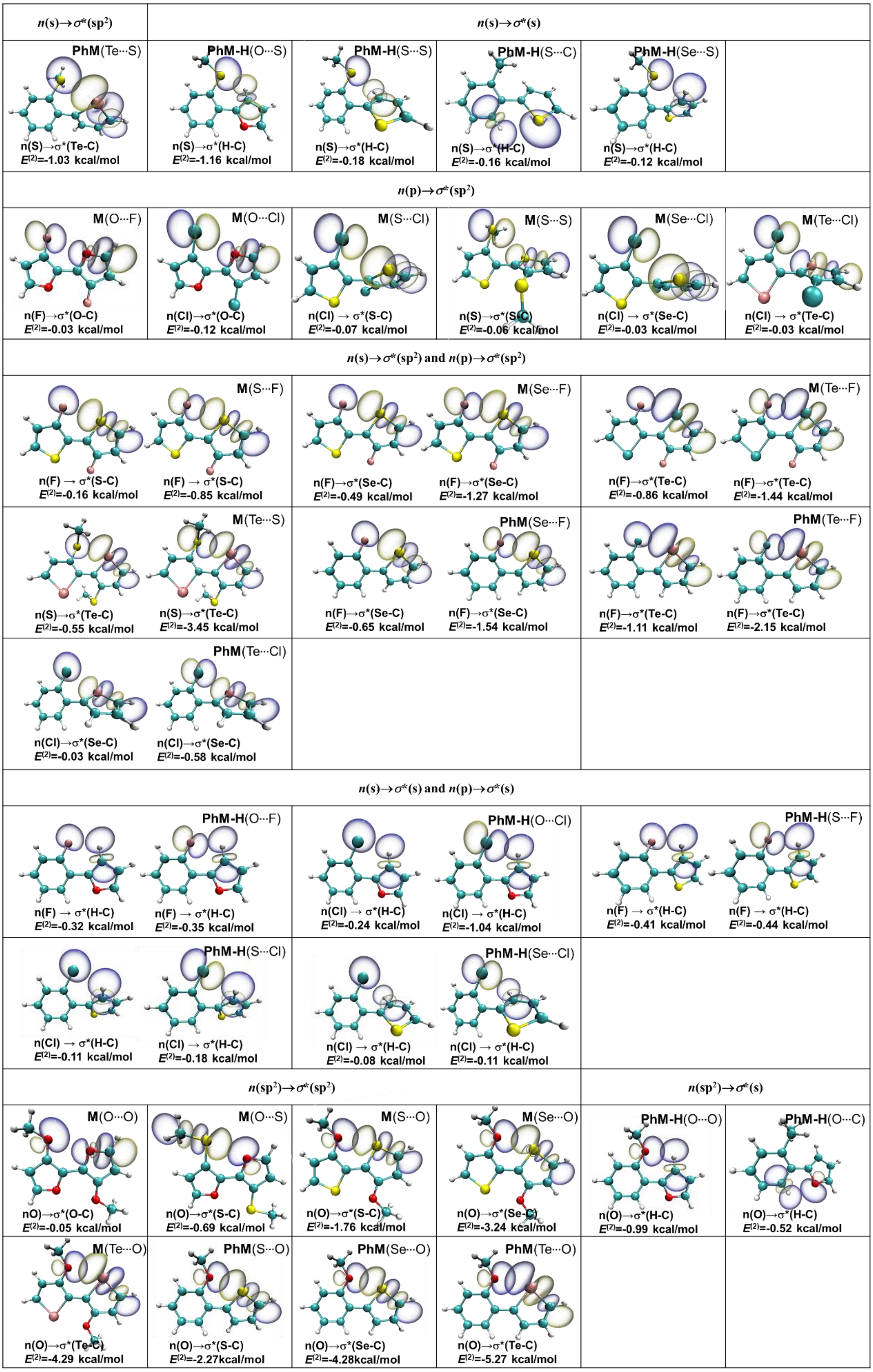

Supplementary Figure S4. NBO overlap diagrams. Classification of four orbital types about

NBO overlap of 36 investigated systems in the gas phase and NBO second-order perturbative energies ($E^{(2)}$).

6. The establishment of the descriptor

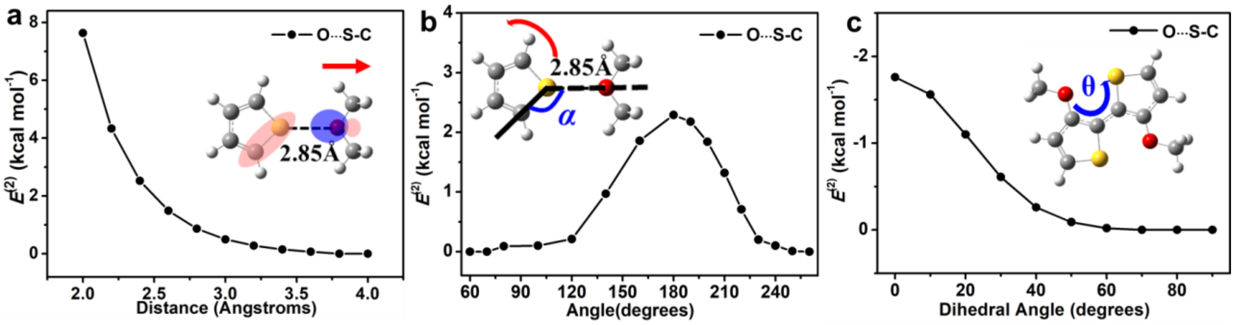

Supplementary Figure S5. Diagram of $E^{(2)}$ distribution with different structural parameters. a Distance, **b** angle between orbitals and **c** dihedral angle and their computational model given as insets.

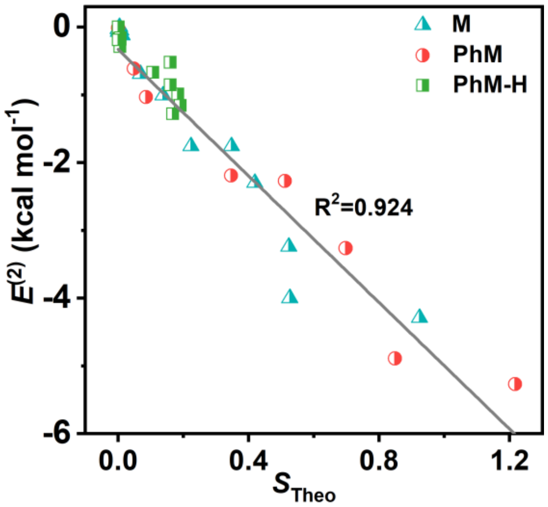

Supplementary Figure S6. Linear relationship between $E^{(2)}$ and S_{Theo} with fitted line. The correlation between $E^{(2)}$ and S ($S=\cos^2\theta\cdot(1-e^{\Delta d})^2$) for 36 compounds at B3LYP(D3)/6-31G+(d) level, which is removing cosα from the S for **PhM-H** systems.

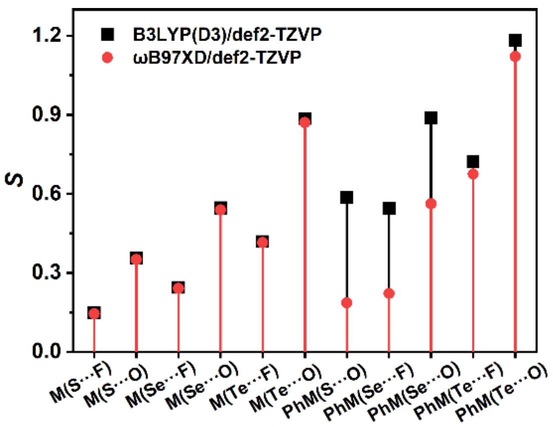

Supplementary Figure S7. Descriptors obtained by different calculation methods. The difference of *S* for 11 compounds which is optimized based on B3LYP(D3)/def2-TZVP and ωB97XD/def2-TZVP level.

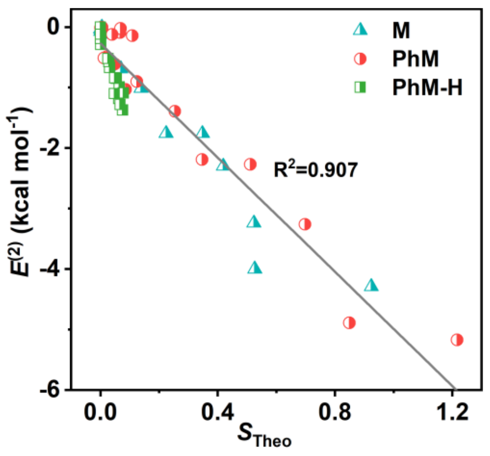

Supplementary Figure S8. The fitted line of $E^{(2)}$ versus S_{Theo}. The correlation between $E^{(2)}$ and *S* for 56 compounds at B3LYP(D3)/6-31G+(d) level.

7. Relaxation energy

Supplementary Table S3. Reorganization energies for **PhM-H**(O···O/C) and **PhM**(X···O/C) (X = S, Se, Te). All the geometrical and electronic structures of ground state and excited state electronic structures in the gas phase are calculated at B3LYP(D3)/6-31+G(d) level.

Systems	λ_{GS} (eV)	λ_{ES} (eV)	λ_{opt} (eV)	λ_{NS} (eV)	λ_{CS} (eV)	λ_{elec} (eV)
**PhM-H**(O···O)	0.143	0.151	0.294	0.149	0.147	0.295

**PhM-H**(O···C)	0.213	0.287	0.500	0.143	0.168	0.311
**PhM(**S···O)	0.153	0.189	0.342	0.157	0.177	0.335
**PhM**(S···C**)**	0.352	0.746	1.098	0.273	0.380	0.653
**PhM**(Se···O)	0.149	0.159	0.308	0.155	0.154	0.309
**PhM**(Se···C)	0.430	0.770	1.200	0.296	0.428	0.724
**PhM**(Te···O)	0.154	0.165	0.319	0.159	0.159	0.318
**PhM**(Te···C)	0.562	0.724	1.287	0.276	0.374	0.650

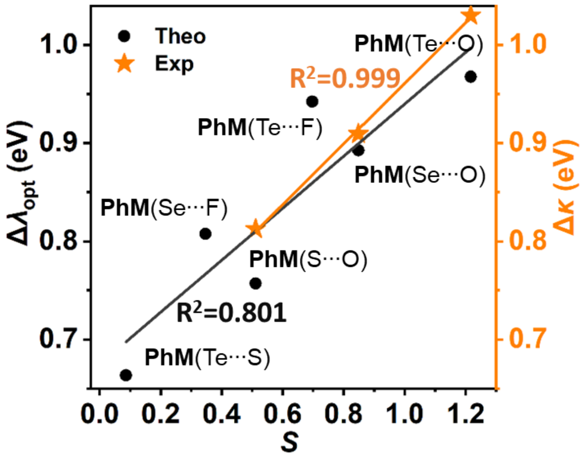

Supplementary Figure S9. The reorganization energy and Stokes shifts with fitted lines. The correlation between calculated $\Delta\lambda_{opt}$ and experimental $\Delta\kappa$ and S for the compounds with noncovalent interactions. $\Delta\lambda_{opt}$ and $\Delta\kappa$ are the differences of the reorganization energy and Stokes shifts between **PhM**(X···Y) and **PhM**(X···C), respectively.

8. The extension of the descriptor

Supplementary Table S4. The descriptor strengths (S) from theoretical optimizing structure in gas and solid and experimental crystal structures of new synthesized molecules.

Systems	S_{Theo} (gas)	S_{Theo} (solid)	S_{Exp}
**PhM-H**(O···O)	0.064	0.086	0.049
**PhM**(S···O)	0.511	0.665	0.769
**PhM**(Se···O)	0.849	1.028	1.084

**PhM**(Te···O)	1.217	1.296	1.419
**PhM**(S···C)	0.003	0.001	0.035
**PhM**(Se···C)	0.001	0.035	0.071

Supplementary Table S5. The dihedral angles (θ), orbital plane angle (α), bond lengths (*d*), descriptor (*S*) and NBO second-order perturbative energy ($E^{(2)}$) for all investigated systems. *d*v is the sum of van der Waals radii of two involved atoms.

Systems	θ (°)	α (°)	*d* (Å)	*d*v (Å)	*S*	$E^{(2)}$ (kcal mol^{-1})	Ref.
**A**	12.42	164.61	2.773	3.32	0.489	-2.20	1[1]
**B**	2.07	169.48	2.783	3.32	0.497	-2.06	2[2]
**C**	6.93	167.30	2.870	3.32	0.311	-1.23	3[3]
**D**	0.82	167.11	3.028	3.32	0.112	-0.81	4[4]
**E**	2.74	175.44	2.669	3.32	0.839	-4.17	5[5]
**F**	104.03	97.86	3.856	3.32	0.001	0.00	5
**G**	0.26	163.68	3.009	3.00	0.000	0.00	6[6]
**H**	0.70	145.55	2.885	3.35	0.289	-1.23	7[7]
**I**	0.88	170.01	2.834	3.32	0.385	-1.49	8[8]
**J**	1.40	167.96	2.926	3.27	0.165	-1.03	9[9]
**K**	0.80	169.78	2.728	3.27	0.509	-1.92	10[10]
**L**	44.89	113.90	2.746	2.99	0.015	-0.01	10

9. Supplementary Notes

The NBO perturbative framework applies qualitative concepts of valence theory to describe the noncovalent energy lowering[11]. NBO protocols partition electron density from diffuse molecular orbitals into localized Lewis-type orbitals, from which the energy of mixing can be computed. As shown in **Supplementary Figure S1**, it depicts the interaction of a filled orbital *n* of the formal Lewis structure with one of the unfilled antibonding orbitals σ* to give the second-order energy lowering ($E^{(2)}$). This energy lowering is given by the formula:

$$E^{(2)} = \Delta E^{(2)}_{n\sigma^*} = -2\frac{\langle n|F|\sigma^*\rangle^2}{\varepsilon_{\sigma^*} - \varepsilon_n} \quad \text{Supplementary Equation (1)}$$

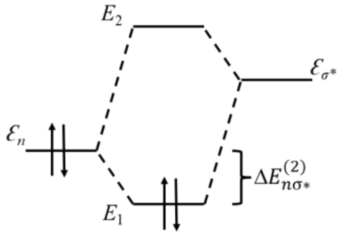

Supplementary Figure S10. The schematic energy diagram. Perturbative donor-acceptor interaction, involving a filled orbital *n* and an unfilled orbital σ*. F is the Fock operator and ε_n and ε_{σ^*} are NBO orbital energies.

10. Supplementary Methods

Proton (^{1}H) NMR, carbon (^{13}C) NMR, selenium (^{77}Se) NMR, tellurium (^{125}Te) NMR and ^{1}H-^{1}H NOESY NMR were measured on a JEOL JNM ECZ500R (500 MHz) nuclear magnetic resonance spectrometer. Chemical shifts are recorded in parts per million (δ ppm) referenced to $CDCl_3$ (δ 7.24 for ^{1}H NMR and δ 77.00 for ^{13}C NMR), $C_2D_2Cl_4$ (δ 5.98 for ^{1}H NMR and δ 73.78 for ^{13}C NMR).

UV-Vis absorption spectra were measured on a Gary 60 UV-Vis Spectrophotometer. All liquid samples were well-dissolved in chloroform. All film samples were spin-coated on glass substrates. X-ray crystallographic data were collected at 113K on the Rigaku mm007 with a

rotating anode and a Saturn70 detector. An exposure time of 3 s and scan width of 1° were set per frames for **PhM**(Se···O). An exposure time of 10 s and scan width of 1° were set per frames for **PhM-H**(S···C). An exposure time of 4 s and scan width of 1° were set per frames for **PhM-H**(O···O). An exposure time of 5 s and scan width of 0.5° were set per frames for **PhM-H**(Se···C). An exposure time of 0.5 s and 1.5 s, and scan width of 0.5° were set per frame for **PhM**(S···O). The structure was solved by the intrinsic phasing method (SHELXT) and refined by the least squares method (SHELXL) integrated in Olex2. X-ray crystallographic data of **PhM**(Te···O) was obtained by D8QUEST X-ray single crystal diffractometer.

Synthesis of molecules:

All reactions were carried out in flame-dried glassware under N_2 atmosphere using standard Schlenk techniques. The toluene, N,N-dimethylformamide and tetrahydrofuran were purified by FLEANO solvent purification systems (FL-MD-5). Reactions were stirred using Teflon-coated magnetic stir bars. Room temperature of 25 ℃ and elevated temperatures were maintained using Thermostat-controlled silicone oil baths. Organic solutions were concentrated using a rotary evaporator with a diaphragm vacuum pump. Analytical TLC was performed on silica gel GF254 plates. The TLC plates were visualized by ultraviolet light (λ = 254 nm and 365 nm). Purification of products was accomplished by column chromatography on silica gel.

The compounds 1,4-dichlorobenzene, hydroquinone, hexylmagnesium bromide (2.0 M), 1-bromohexane, iodine, iodic acid, sulfuric acid (98 wt%), acetic acid (37 wt%), tellurium granules, furan, tributyl (thiophen-2-yl) stannane and tributyl (selenophen-2-yl) stannane were purchased from various commercial sources and used without further purification. Attention should be paid while handling organotellurium, selenium compounds, sulfuric acid, and acetic acid. The 1,4-dihexyl-2,5-diiodobenzene, 1,4-bis(hexyloxy)-2,5-diiodobenzene, tributyl(tellurophen-2-yl)stannane, and tributyl(furan-2-yl)stannane were prepared according to reported literatures and listed below. [2,3]

1, 4-dihexylbenzene

Under nitrogen, 1 equiv of 1,4-dichlorobenzene (1.00g, 6.80 mmol) and $NiCl_2$(dppp) (dppp = 1,3-bis(diphenylphosphino)propane) (12 mg, 0.02 mmol) was slowly added to a solution of alkylmagnesiumbromide (1M in THF, 20 ml) in THF (10 ml). The reaction mixture was refluxed for 48 h. The mixture is poured onto ice, diluted HCl was added, the organic layer was separated and washed neutral with H_2O (3 × 100 mL), and the aqueous layer was extracted with DCM. The combined organic layers were dried over MgSO4. After evaporation of solvent the colorless oil was directly exerted in the next step.

1,4-dihexyl-2,5-diiodobenzene

To a solution of 1,4-dihexylbenzene (5.00 g, 18.00 mmol), 90 mL of acetic acid, 3 mL of water, and 6 mL of concentrated H_2SO_4, HIO_3 (1.60 g, 9.00 mmol) and I_2 (5.08 g, 19.80 mmol) were added. The reaction mixture was stirred at 115°C for 5 h and refluxed, and then cooled to room temperature. After most of the acetic acid was evaporated under reduced pressure, aqueous Na_2SO_3 (20%) was added to quench excess iodine. The mixture was poured into ice water and extracted with DCM (3×100 mL). After washed with water and brine and dried over $MgSO_4$, the crude product was filtered through a short silica gel column using PE as eluent, recrystallized from EtOH (60 mL) and dried in vacuo. The product was received as a white powder (2.11 g, 22 %). ^{1}H-NMR (500 MHz, $CDCl_3$): δ = 7.59 (s, 2 H), 2.59 (t, ^{3}J(H,H) = 7.8 Hz, 4 H), 1.58-1.46 (m, 4 H), 1.37-1.28 (m, 12 H), 0.90 (t, ^{3}J(H,H) = 6.8 Hz, 6 H) ppm; ^{13}C-NMR (100 MHz, $CDCl_3$): δ =144.69, 139.15, 100.18, 39.70, 31.46, 30.03, 28.85, 22.45, 13.94.

1,4-bis(hexyloxy)benzene

A 100 mL flask was equipped with a stir bar. Sequentially, hydroquinone (2.00 g, 0.02 mol), anhydrous DMF (20 mL), potassium carbonate (6.26 g, 0.05 mol), and 1- bromohexane (7.46 g, 0.05 mol) were added to the flask. The reaction mixture was stirred vigorously under N_2 at

80 °C for 3 d. After cooling to rt, the reaction mixture was poured into water (100 mL). The mixture was extracted with hexanes (3 x 100 mL). The organic layer was washed with water (2 x 100 mL) and brine (1 x 100 mL), then dried over anhydrous $MgSO_4$, filtered, and concentrated in vacuo. Crude product was obtained as red oil, which was directly exerted in the next step without purification.

1,4-bis(hexyloxy)-2,5-diiodobenzene

To a solution of 1,4-bis(hexyloxy)benzene (1.67 g, 6.8 mmol), 30 mL of acetic acid, 1 mL of water, and 2 mL of concentrated H_2SO_4, HIO_3 (0.60 g, 3.4 mmol) and I_2 (1.90 g, 7.5 mmol) were added. The reaction mixture was stirred at 115°C for 5 h and refluxed, and then cooled to room temperature. After most of the acetic acid was evaporated under reduced pressure, aqueous Na_2SO_3 (20%) was added to quench excess iodine. The mixture was poured into ice water and extracted with DCM (3×100 mL). After washed with water and brine and dried over $MgSO_4$, the crude product was filtered through a short silica gel column using PE as eluent, recrystallized from EtOH (60 mL) and dried in vacuo. The product was received as a white powder (2.5 g, 74 %). ^{1}H NMR (500 MHz, CDCl3): δ =7.17 (s, 2H), 3.92 (t, 4H), 1.84-1.75 (m, 4H), 1.55-1.34 (m, 12H), 0.91 (t, 6H). ^{13}C NMR (100 MHz, CDCl3): δ =152.9, 122.8, 86.3, 70.3, 31.4, 29.1, 25.7, 22.5, 14.0.

Tellurophene

Tellurium granules (4 g, 31.5 mmol) and $NaBH_4$ (5.2 g, 140.4 mmol) were blended in a 250mL flask (flask A) and purged with N_2. Distilled water (60 mL) was added and the mixture was stirred. In a second 250 mL flask (flask B), fitted with a condenser, a solution of 15.6 g (0.276 mol) KOH in 60 mL water was prepared and brought to reflux. When tellurium reduction in flask A was complete (no visible tellurium granules and hydrogen gas evolution remained) the

resultant violet solution was cooled in an ice bath and 60 mL of degassed MeOH was added. At this point, a cannula was fitted to the top of the condenser on flask B and connected to flask A. A solution of 9.6 mL of 1,4-dichloro-2-butyne in 12.4 mL of 1,4-dioxane was added in portions (~15 min) to flask B, and the resulting gas (diacetylene) was allowed to bubble into the sodium telluride solution in flask A. The ice bath was immediately removed and the reaction was allowed to proceed for 1 hour with constant stirring. The mixture in flask A was diluted with hexanes (30 mL), quenched with brine (20 mL), and filtered through Celite. The aqueous layer was extracted with hexanes, and the organic layer was washed with brine, dried over $MgSO_4$, and the solvent was removed by evaporation. The resultant brown oil was filtered through a silica gel plug to acquire 2.55 g (45%) of the title compound, dark orange oil.

tributyl(tellurophen-2-yl)stannane

A solution of n-BuLi (5.90 mL, 2.5 M in hexanes) was added dropwise to a solution of tellurophene (1 g, 15 mmol) in dry tetrahydrofuran (THF, 10 mL) at -78°C under N_2. The reaction mixture was allowed to reach 0 °C and stirred for 1 h. The resulting mixture was cooled to -78°C, Bu_3SnCl (4.78 g, 15 mmol) was added dropwise, and the reaction mixture was allowed to reach room temperature and stirred overnight. The mixture was quenched with water, extracted with DCM, dried with $MgSO_4$, and evaporated. Brown oil was acquired without further purification.

tributyl(furan-2-yl)stannane

A solution of n-BuLi (5.90 mL, 2.5 M in hexanes) was added dropwise to a solution of furan (1 g, 15 mmol) in dry tetrahydrofuran (THF, 10 mL) at -78°C under N_2. The reaction mixture was allowed to reach 0 °C and stirred for 1 h. The resulting mixture was cooled to -78 °C, Bu_3SnCl (4.78 g, 15 mmol) was added dropwise, and the reaction mixture was allowed to reach

room temperature and stirred overnight. The mixture was quenched with water, extracted with DCM, dried with $MgSO_4$, and evaporated. Yellow oil was acquired without further purification.

2,2'-(2,5-bis(hexyloxy)-1,4-phenylene)bis(furan) **(PhM-H**(O···O)**)**

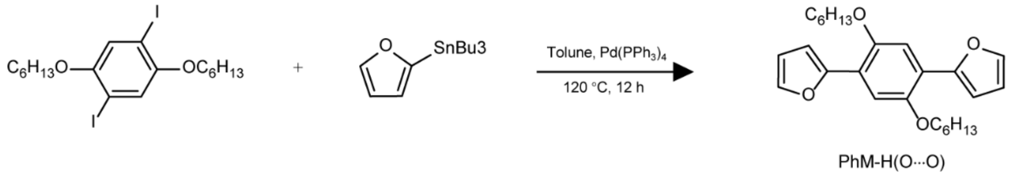

Supplementary Figure S11. Synthesis of **PhM-H**(O···O)

In a round-bottom flask (100 mL), 1,4-dihexyloxy-2,5-diiodobenzene (381mg, 0.72 mmol), tributyl(furan-2-yl)stannane (500 mg, 2.2 mmol) and $Pd(PPh_3)_4$ (41.5 mg, 0.04 mmol) were added under nitrogen in anhydrous toluene (20.0 mL). The mixture was stirred and refluxed at 120 °C for 12 h. Afterwards, an aqueous potassium fluoride solution (3.0 M, 40.0 mL) was added to the mixture. After quenching the reaction, the organic phase was extracted with dichloromethane (3 × 100 mL). The combined organic portion was collected, washed with water and brine, and dried over $MgSO_4$. After filtration, the solution was filtered through a short silica gel column (petroleum ether), concentrated to afford the crude product, which is subjected to the recrystallization in hexane. Finally, the crystals were dried in vacuo and isolated as an off-white solid (155.0 mg, 53% yield). ^{1}H NMR (500 MHz, Chloroform-*d*) δ 7.47 (d, J = 1.0 Hz, 1H), 7.44 (s, 1H), 7.03 (d, J = 3.3 Hz, 1H), 6.51 (dd, J = 3.4, 1.8 Hz, 1H), 4.13 (t, J = 6.6 Hz, 2H), 2.00 – 1.84 (m, 2H), 1.53 (dd, J = 14.1, 8.4 Hz, 2H), 1.38 (dt, J = 9.1, 4.2 Hz, 5H), 0.93 (d, J = 7.0 Hz, 3H). ^{13}C NMR (126 MHz, Chloroform-*d*) δ 150.33, 148.95, 140.96, 118.75, 111.99, 110.23, 109.35, 68.91, 31.62, 29.47, 29.33, 25.95, 22.59, 14.04. MS (LC-MS): Calcd for $C_{26}H_{34}O_4$ $[M+Na]^+$ 433.2349; found: 433.2349.

2,2'-(2,5-bis(hexyloxy)-1,4-phenylene)bis(thiophene) **(PhM**(S···O)**)**

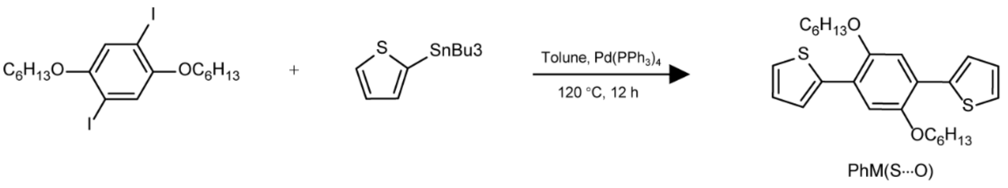

Supplementary Figure S12. Synthesis of **PhM**(S···O)

In a round-bottom flask (100 mL), 1,4-dihexyloxy-2,5-diiodobenzene (270 mg, 0.51 mmol), tributyl(thiophene-2-yl)stannane (500 mg, 1.34 mmol) and $Pd(PPh_3)_4$ (80 mg, 0.07 mmol) were added under nitrogen in anhydrous toluene (20.0 mL). The mixture was stirred and refluxed at 120 °C for 12 h. Afterwards, an aqueous potassium fluoride solution (3.0 M, 40.0 mL) was added to the mixture. After quenching the reaction, the organic phase was extracted with dichloromethane (3 × 100 mL). The combined organic portion was collected, washed with water and brine, and dried over $MgSO_4$. After filtration, the solution was filtered through a short silica gel column (petroleum ether), concentrated to afford the crude product, which is subjected to the recrystallization in hexane. Finally, the crystals were dried in vacuo and isolated as a yellow solid (140.0 mg, 63 % yield). ^{1}H NMR (500 MHz, Tetrachloroethane-d_2) δ 7.55 (d, *J* = 2.3 Hz, 1H), 7.36 (d, *J* = 6.4 Hz, 1H), 7.25 (s, 1H), 7.14 – 7.09 (m, 1H), 4.08 (t, *J* = 6.6 Hz, 2H), 1.95 – 1.85 (m, 2H), 1.53 (t, *J* = 6.4 Hz, 2H), 1.35 (q, *J* = 4.1, 3.6 Hz, 4H), 0.99 – 0.85 (m, 3H). ^{13}C NMR (126 MHz, Tetrachloroethane-d_2) δ 149.23, 139.23, 127.05, 126.19, 125.37, 122.86, 112.60, 69.89, 31.77, 29.53, 26.09, 22.82, 14.38. MS (LC-MS): Calcd for $C_{26}H_{34}S_2O_2$ $[M+H]^+$ 443.2073; found: 443.2070.

2,2'-(2,5-bis(hexyloxy)-1,4-phenylene)bis(selenophene) **(PhM**(Se···O)**)**

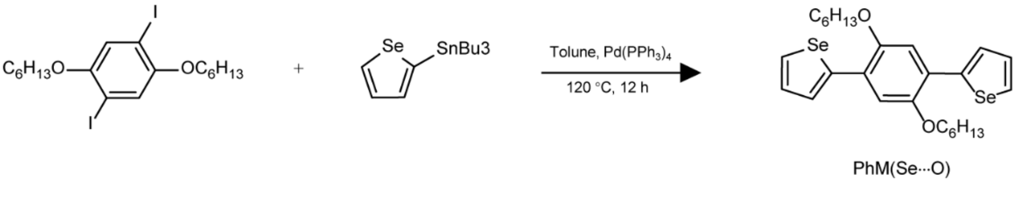

Supplementary Figure S13. Synthesis of **PhM**(Se···O)

In a round-bottom flask (100 mL), 1,4-dihexyloxy-2,5-diiodobenzene (220 mg, 0.42 mmol), tributyl(selenophene-2-yl)stannane (500 mg, 1.19 mmol) and $Pd(PPh_3)_4$ (120 mg, 0.1 mmol) were added under nitrogen in anhydrous toluene (20.0 mL). The mixture was stirred and refluxed at 120 °C for 12 h. Afterwards, an aqueous potassium fluoride solution (3.0 M, 40.0 mL) was added to the mixture. After quenching the reaction, the organic phase was extracted with dichloromethane (3 × 100 mL). The combined organic portion was collected, washed with water and brine, and dried over $MgSO_4$. After filtration, the solution was filtered through a short silica gel column (petroleum ether), concentrated to afford the crude product, which is subjected to the recrystallization in hexane. Finally, the crystals were dried in vacuo and isolated as a yellow solid (82.0 mg, 37% yield). ^{1}H NMR (500 MHz, Tetrachloroethane-d_2) δ 8.08 (d, J = 5.6 Hz, 1H), 7.73 (d, J = 3.7 Hz, 1H), 7.37 (t, J = 4.8 Hz, 1H), 7.32 (s, 1H), 4.12 (t, J = 6.6 Hz, 2H), 2.02 – 1.87 (m, 2H), 1.54 (t, J = 7.6 Hz, 2H), 1.34 (d, J = 10.8 Hz, 4H), 0.91 (t, J = 7.0 Hz, 3H). ^{13}C NMR (126 MHz, Tetrachloroethane-d_2) δ 148.58, 142.89, 132.42, 129.18, 126.07, 124.46, 110.78, 69.98, 31.81, 29.61, 26.19, 22.83, 14.41. ^{77}Se NMR (95 MHz, Tetrachloroethane-d_2) δ 645.70. MS (LC-MS): Calcd for $C_{26}H_{34}Se_2O_2$ $[M+H]^+$ 537.0970; found: 537.0964.

2,2'-(2,5-bis(hexyloxy)-1,4-phenylene)bis(tellurophene) **(PhM**(Te···O)**)**

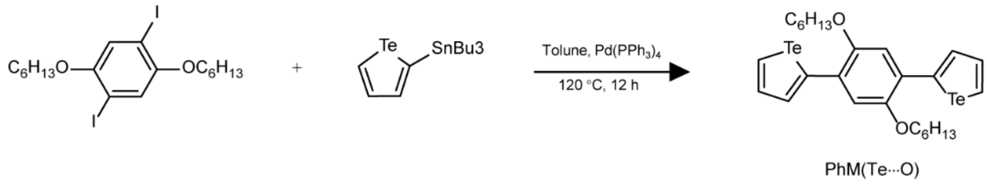

Supplementary Figure S14. Synthesis of **PhM**(Te···O)

In a round-bottom flask (100 mL), 1,4-dihexyloxy-2,5-diiodobenzene (153 mg, 0.29 mmol), tributyl(tellurophene-2-yl)stannane (300 mg, 0.87 mmol) and $Pd(PPh_3)_4$ (24 mg, 0.03 mmol) were added under nitrogen in anhydrous toluene (20.0 mL). The mixture was stirred and

refluxed at 120 °C for 12 h. Afterwards, an aqueous potassium fluoride solution (3.0 M, 40.0 mL) was added to the mixture. After quenching the reaction, the organic phase was extracted with dichloromethane (3 × 100 mL). The combined organic portion was collected, washed with water and brine, and dried over $MgSO_4$. After filtration, the solution was filtered through a short silica gel column (petroleum ether), concentrated to afford the crude product, which is subjected to the recrystallization in hexane. Finally, the crystals were dried in vacuo and isolated as a yellow solid (69.0 mg, 38 % yield). ^{1}H NMR (500 MHz, Tetrachloroethane-d_2) δ 9.00 (d, J = 7.0 Hz, 1H), 8.17 (d, J = 4.3 Hz, 1H), 7.95 (dd, J = 7.0, 4.2 Hz, 1H), 7.43 (s, 1H), 4.20 (t, J = 6.9 Hz, 2H), 2.01 (t, J = 7.5 Hz, 2H), 1.63 – 1.51 (m, 2H), 1.38 (t, J = 8.0 Hz, 4H), 0.92 (d, J = 6.5 Hz, 3H). ^{13}C NMR (126 MHz, Tetrachloroethane-d_2) δ 136.76, 131.99, 129.95, 108.36, 74.31, 74.09, 73.87, 70.11, 31.81, 29.75, 26.33, 22.84, 14.40. ^{125}Te NMR (158 MHz, Tetrachloroethane-d_2) δ 851.62. MS (LC-MS): Calcd for $C_{26}H_{34}Te_2O_2$ $[M]^+$ 638.0678; found: 638.0680.

2,2'-(2,5-dihexyl-1,4-phenylene)bis(furan) (**PhM**(O···C)**)**

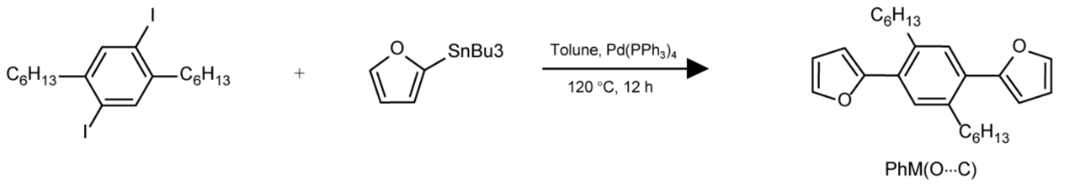

Supplementary Figure S15. Synthesis of **PhM**(O···C)

In a round-bottom flask (100 mL), 1,4-dihexyl-2,5-diiodobenzene (450 mg, 1.11 mmol), tributyl(furan-2-yl)stannane (652 mg, 2.81 mmol) and $Pd(PPh_3)_4$ (200 mg, 0.17 mmol) were added under nitrogen in anhydrous toluene (20.0 mL). The mixture was stirred and refluxed at 120 °C for 12 h. Afterwards, an aqueous potassium fluoride solution (3.0 M, 40.0 mL) was added to the mixture. After quenching the reaction, the organic phase was extracted with dichloromethane (3 × 100 mL). The combined organic portion was collected, washed with

water and brine, and dried over $MgSO_4$. After filtration, the solution was filtered through a short silica gel column (petroleum ether), concentrated to afford the crude product, which is subjected to the recrystallization in hexane. Finally, the crystals were dried in vacuo and isolated as a yellow solid (216.0 mg, 63% yield). ^{1}H NMR (500 MHz, Tetrachloroethane-d_2) δ 7.55 (d, J = 1.7 Hz, 1H), 7.53 (s, 1H), 6.58 – 6.50 (m, 2H), 2.87 – 2.74 (m, 2H), 1.70 – 1.54 (m, 2H), 1.41 (t, J = 7.3 Hz, 2H), 1.36 – 1.28 (m, 4H), 0.97 – 0.83 (m, 3H). ^{13}C NMR (126 MHz, Tetrachloroethane-d_2) δ 153.53, 142.05, 137.40, 129.64, 129.07, 111.70, 108.50, 34.16, 31.88, 30.99, 29.94, 29.65, 22.86, 14.42. MS (LC-MS): Calcd for $C_{26}H_{34}O_2$ $[M+H]^+$ 379.2632; found: 379.2630.

2,2'-(2,5-dihexyl-1,4-phenylene)bis(thiophene) **(PhM**(S···C)**)**

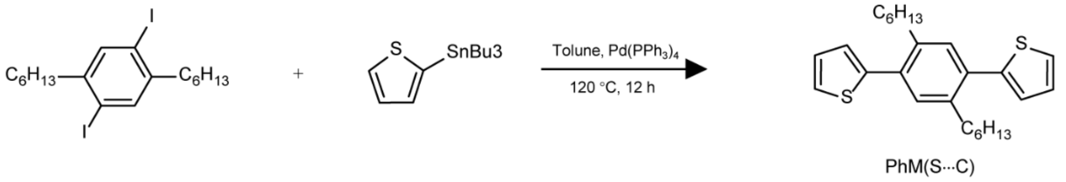

Supplementary Figure S16. Synthesis of **PhM**(O···C)

In a round-bottom flask (100 mL), 1,4-dihexyl-2,5-diiodobenzene (215 mg, 0.53 mmol), tributyl(thiophene-2-yl)stannane (500 mg, 1.34 mmol) and $Pd(PPh_3)_4$ (65 mg, 0.06 mmol) were added under nitrogen in anhydrous toluene (20.0 mL). The mixture was stirred and refluxed at 120 °C for 12 h. Afterwards, an aqueous potassium fluoride solution (3.0 M, 40.0 mL) was added to the mixture. After quenching the reaction, the organic phase was extracted with dichloromethane (3 × 100 mL). The combined organic portion was collected, washed with water and brine, and dried over $MgSO_4$. After filtration, the solution was filtered through a short silica gel column (petroleum ether), concentrated to afford the crude product, which is subjected to the recrystallization in hexane. Finally, the crystals were dried in vacuo and isolated as a white solid (200.0 mg, 91 % yield).^{1}H NMR (500 MHz, Tetrachloroethane-d_2) δ 7.37 (dd, J =

5.2, 1.2 Hz, 1H), 7.28 (s, 1H), 7.11 (dd, J = 5.1, 3.5 Hz, 1H), 7.07 (dd, J = 3.5, 1.2 Hz, 1H), 2.85 – 2.49 (m, 2H), 1.55 (p, J = 7.6 Hz, 2H), 1.30 (p, J = 7.0 Hz, 2H), 1.23 (h, J = 4.6, 4.1 Hz, 4H), 0.85 (t, J = 6.8 Hz, 3H). ^{13}C NMR (126 MHz, Tetrachloroethane-d_2) δ 142.81, 138.60, 133.44, 132.30, 127.38, 126.62, 125.47, 33.27, 31.75, 31.64, 29.44, 22.77, 14.37. MS (LC-MS): Calcd for $C_{26}H_{34}S_2$ $[M+H]^+$ 411.2175; found: 411.2169.

2,2'-(2,5-dihexyl-1,4-phenylene)bis(selenophene) **(PhM**(Se···C)**)**

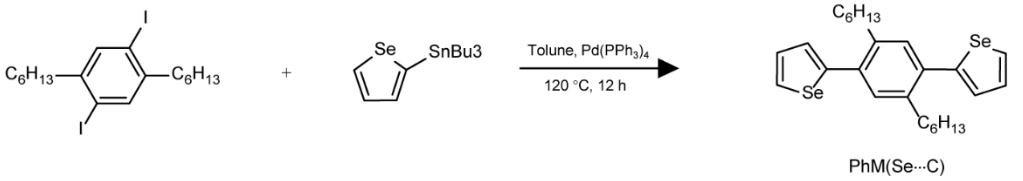

Supplementary Figure S17. Synthesis of **PhM**(Se···C)

In a round-bottom flask (100 mL), 1,4-dihexyl-2,5-diiodobenzene (190 mg, 0.47 mmol), tributyl(selenophene-2-yl)stannane (500 mg, 1.19 mmol) and $Pd(PPh_3)_4$ (60 mg, 0.05 mmol) were added under nitrogen in anhydrous toluene (20.0 mL). The mixture was stirred and refluxed at 120 °C for 12 h. Afterwards, an aqueous potassium fluoride solution (3.0 M, 40.0 mL) was added to the mixture. After quenching the reaction, the organic phase was extracted with dichloromethane (3 × 100 mL). The combined organic portion was collected, washed with water and brine, and dried over $MgSO_4$. After filtration, the solution was filtered through a short silica gel column (petroleum ether), concentrated to afford the crude product, which is subjected to the recrystallization2 in hexane. Finally, the crystals were dried in vacuo and isolated as a white solid (166.0 mg, 69 % yield). ^{1}H NMR (500 MHz, Tetrachloroethane-d_2) δ 8.05 (d, J = 6.8 Hz, 1H), 7.34 (dd, J = 5.7, 3.7 Hz, 1H), 7.26 (s, 1H), 7.20 (d, J = 3.6 Hz, 1H), 2.75 – 2.63 (m, 2H), 1.55 (dt, J = 15.5, 7.6 Hz, 2H), 1.34 – 1.20 (m, 6H), 0.85 (t, J = 6.7 Hz, 3H). ^{13}C NMR (126 MHz, Tetrachloroethane-d_2) δ 149.13, 138.09, 135.44, 132.26, 131.22, 129.91, 128.83,

54.00, 33.27, 31.77, 31.66, 29.45, 22.79, 14.40. ^{77}Se NMR (95 MHz, Tetrachloroethane-d_2) δ 638.62. MS (LC-MS): Calcd for $C_{26}H_{34}Se_2$ $[M+H]^+$ 509.1070; found: 507.1065.

2,2'-(2,5-dihexyl-1,4-phenylene)bis(tellurophene) **(PhM**(Te···C)**)**

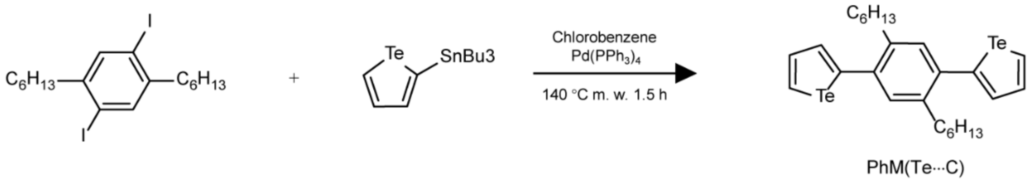

Supplementary Figure S18. Synthesis of **PhM**(Te···C)

In a microwave tube, 1,4-dihexyl-2,5-diiodobenzene (100.2 mg, 0.2 mmol), 2-tributyl(tellurophene-2-yl)stannane (271.4 mg, 0.58 mmol) and $Pd(PPh_3)_4$ (15.6 mg, 0.01 mmol) were added under nitrogen in 5 mL of anhydrous chlorobenzene. The mixture was heated at 140 °C under microwave condition for 1.5 hours. Afterwards, an aqueous potassium fluoride solution (3 M, 20 mL) was added into the solution. After quenching organotin compounds, the organic phase was extracted with DCM (3 × 50 mL), filtered, and washed with water and brine and dried over $MgSO_4$. The crude product was filtered through a short silica gel column (PE as eluent) to afford a yellow cream-like solid (50 mg, 41% yield). ^{1}H NMR (500 MHz, Chloroform-d) δ 8.95 (d, J = 6.8 Hz, 1H), 7.81 (dd, J = 7.0, 3.8 Hz, 1H), 7.49 (d, J = 3.9 Hz, 1H), 7.22 (s, 1H), 2.69 (t, J = 8.0 Hz, 2H), 1.34 – 1.19 (m, 10H), 0.85 (t, J = 6.7 Hz, 4H). ^{13}C NMR (126 MHz, Chloroform-d) δ 147.33, 139.05, 137.49, 137.22, 136.82, 131.80, 127.12, 33.24, 31.68, 31.39, 29.36, 22.64, 14.16. ^{125}Te NMR (158 MHz, Tetrachloroethane-d_2) δ 841.78. MS (LC-MS): Calcd for $C_{26}H_{34}Te_2$ $[M+H]^+$ 607.0858; found: 607.0853.

11. The experimental characterizations of NoCLs

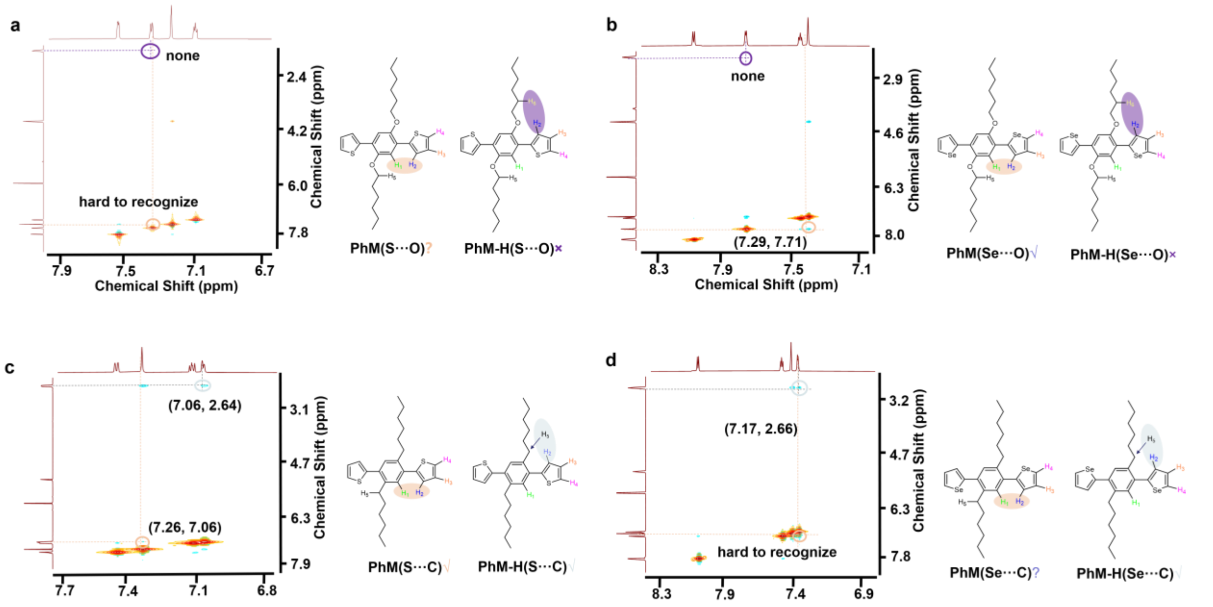

Supplementary Figure S19. ^{1}H-^{1}H NOESY Spectra and possible structures of a PhM(S···O); b PhM(Se···O); c PhM(S···C); d PhM(Se···C). Relevant NOESY interaction signals are circled as brown between H_2 (marked as blue) and H_1 (marked as green) in **PhM**(X···Y) (X= S, Se; Y= O, C); purple between H_2 (marked as blue) and H_6 (marked as yellow) in **PhM-H**(S···O) and **PhM-H**(Se···O); and silver between H_2 (marked as blue) and H_5 (marked as black) in **PhM-H**(S···C) and **PhM-H**(Se···C). Coordinates of the signals are displayed in black brackets, and missing signals are shown as "none".

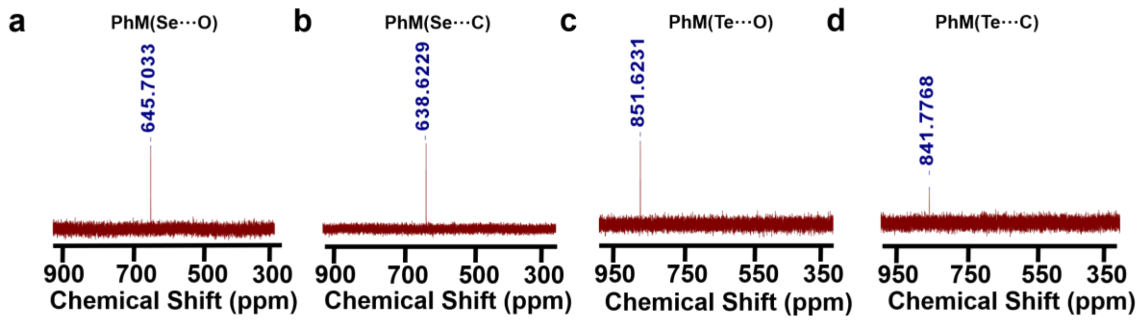

Supplementary Figure S20. **^{77}Se and ^{125}Te NMR of a PhM(Se···O) and b PhM(Se···C) and ^{125}Te NMR of c PhM(Te···O) and d PhM(Te···C).** All signals are marked with blue figures.

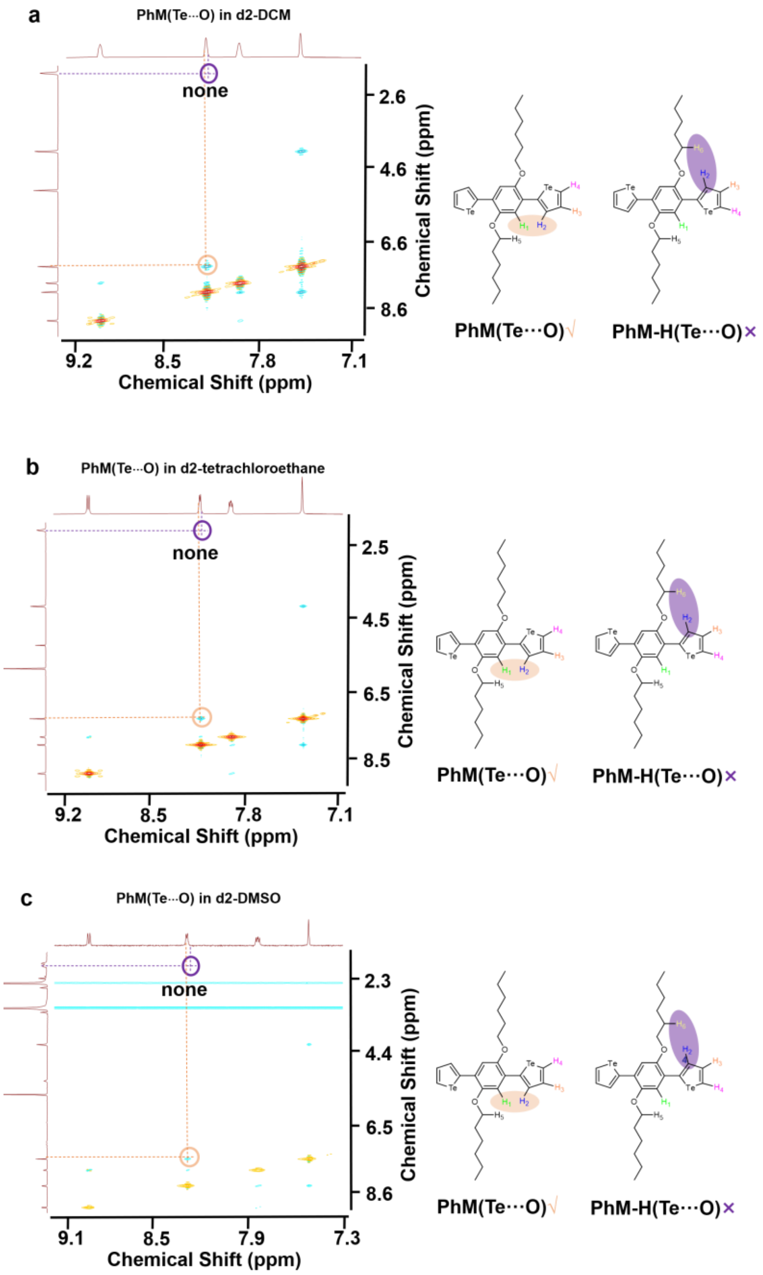

Supplementary Figure S21. ^{1}H-^{1}H NOESY spectra of PhM(Te···O) in different solutions of a d2-DCM, b d2-tetrachloroethane, c d2-DMSO. Relevant NOESY interaction signals are circled as brown between H_2 (marked as blue) and H_1 (marked as green) in **PhM**(Te···O); and purple between H_2 (marked as blue) and H_6 (marked as yellow) in **PhM-H**(Te···O). Coordinates of the signals are displayed in black brackets, and missing signals are shown as “none”.

12. UV–Vis spectra

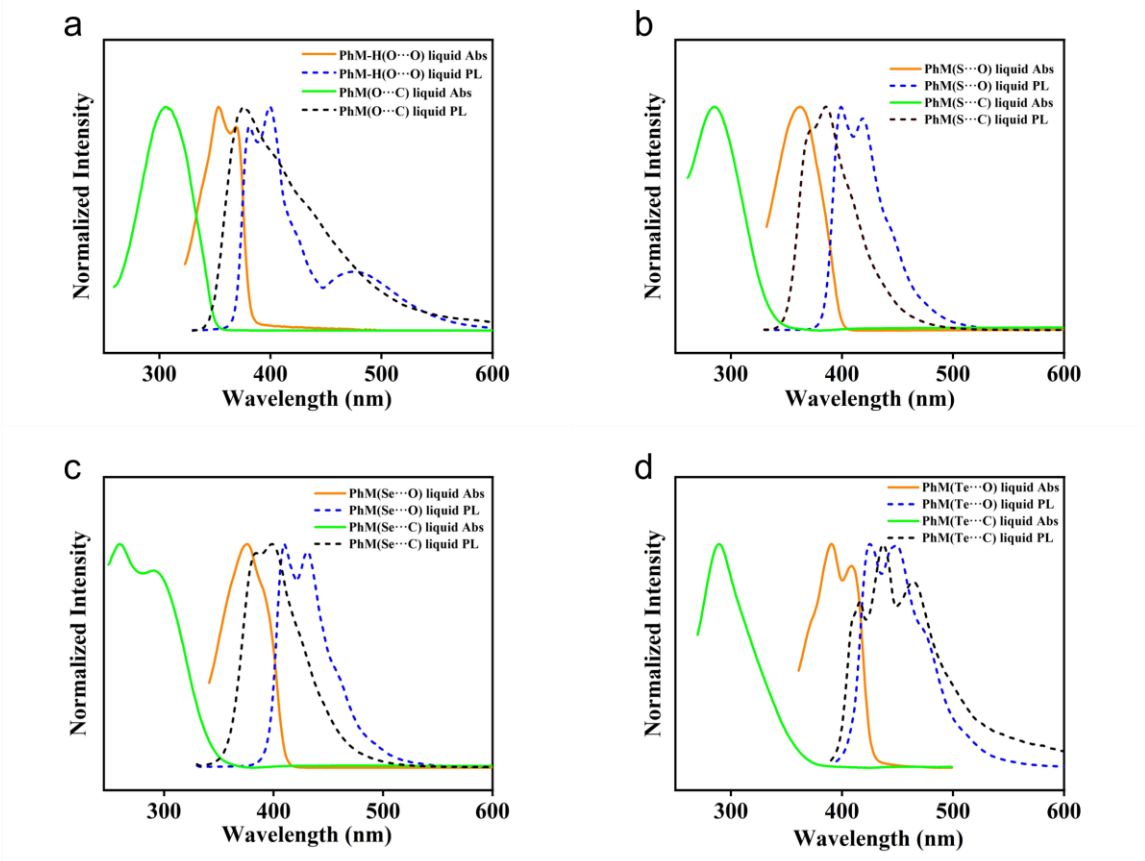

Supplementary Figure S22. Absorption and emission spectra of a PhM-H(O···O/C) and b, c, d PhM(X···O/C) (X = S, Se, Te) in Trichloromethane Solution. Curves of different states and compounds are distinguished by orange and green full lines, as well as blue and black dot lines.

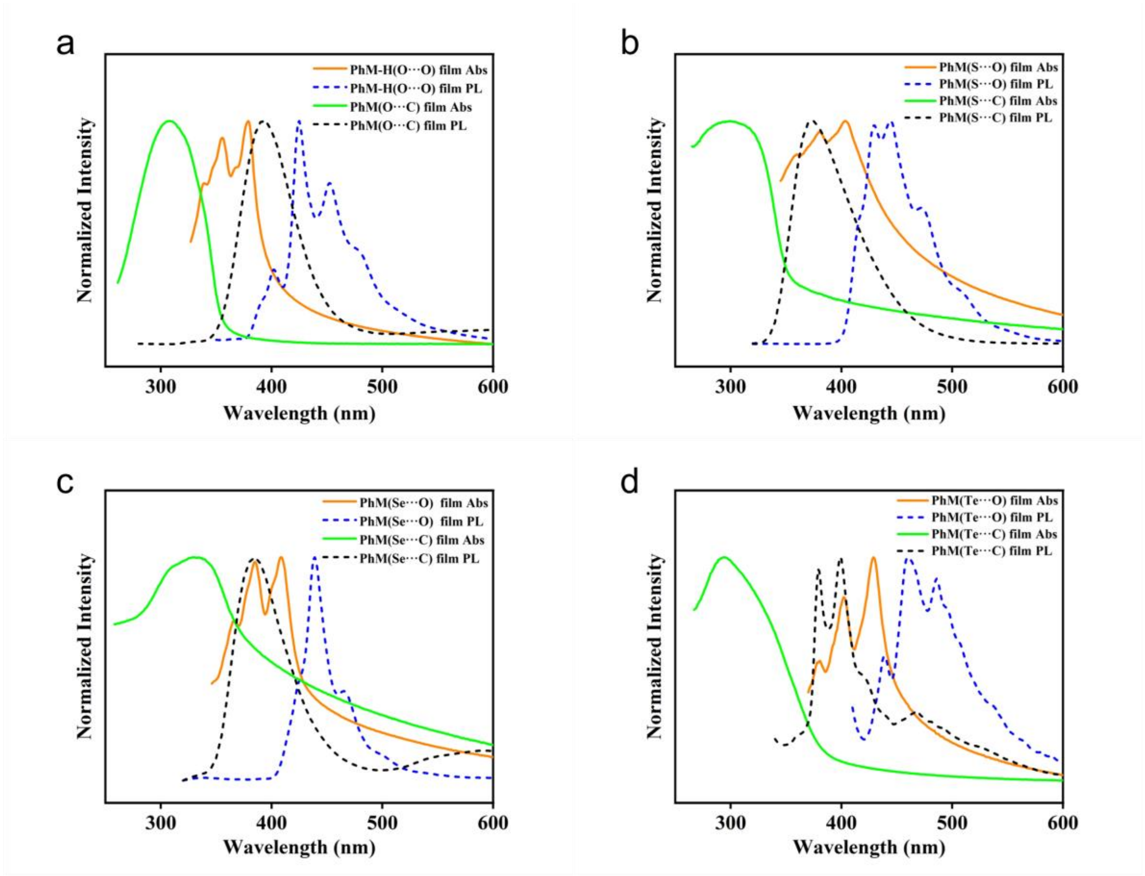

Supplementary Figure S23. Absorption and emission spectra of a PhM-H(O···O/C) and b, c, d PhM(X···O/C) (X = S, Se, Te) in Film State. Curves of different states and compounds are distinguished by orange and green full lines, as well as blue and black dot lines.

Supplementary Table S6. κ and $\Delta\kappa$ in different solvents of chloroform, THF and tolune.

<table>
<tr><th rowspan="2">Systems</th><th colspan="2">Chloroform</th><th colspan="2">THF</th><th colspan="2">Tolune</th></tr>
<tr><th>κ(nm)</th><th>Δκ (nm)</th><th>κ(nm)</th><th>Δκ (nm)</th><th>κ(nm)</th><th>Δκ (nm)</th></tr>
<tr><td>PhM(S···O)</td><td>37</td><td rowspan="2">63</td><td>33</td><td rowspan="2">66</td><td>35</td><td rowspan="2">56</td></tr>
<tr><td>PhM(S···C)</td><td>100</td><td>99</td><td>91</td></tr>
<tr><td>PhM(Se···O)</td><td>34</td><td rowspan="2">66</td><td>32</td><td rowspan="2">76</td><td>36</td><td rowspan="2">75</td></tr>
<tr><td>PhM(Se···C)</td><td>100</td><td>108</td><td>111</td></tr>
</table>

13. NMR spectra and Mass spectrometry

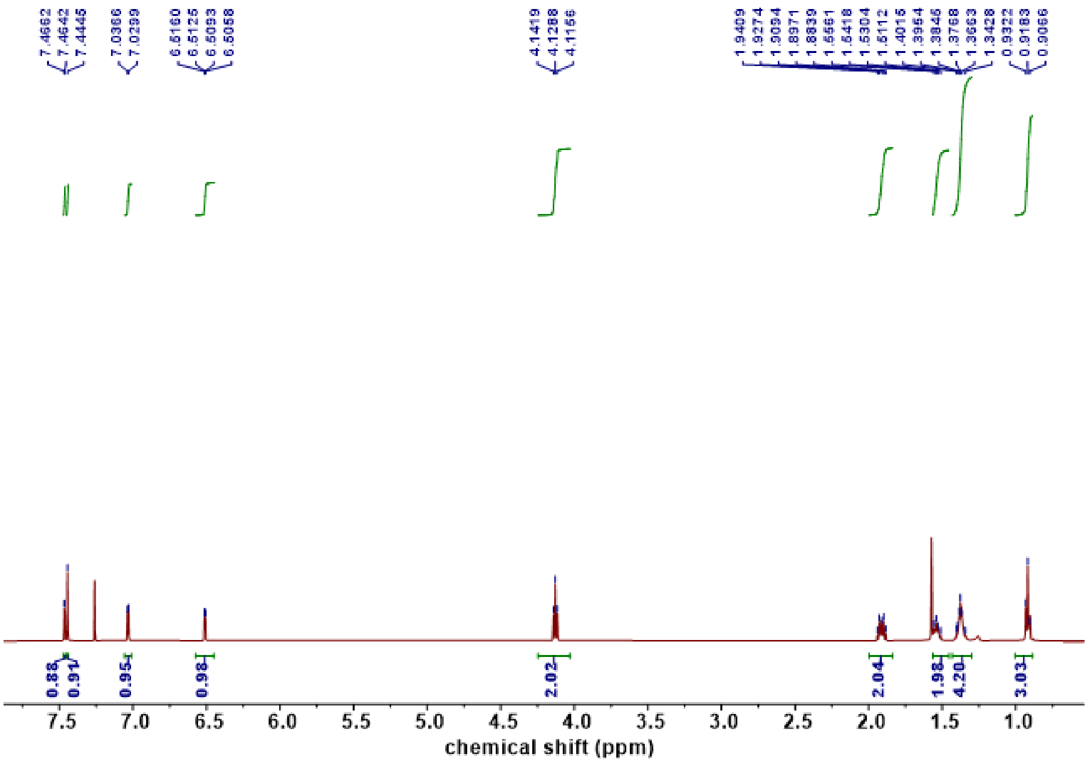

Supplementary Figure S24. ^{1}H NMR of **PhM-H**(O···O)

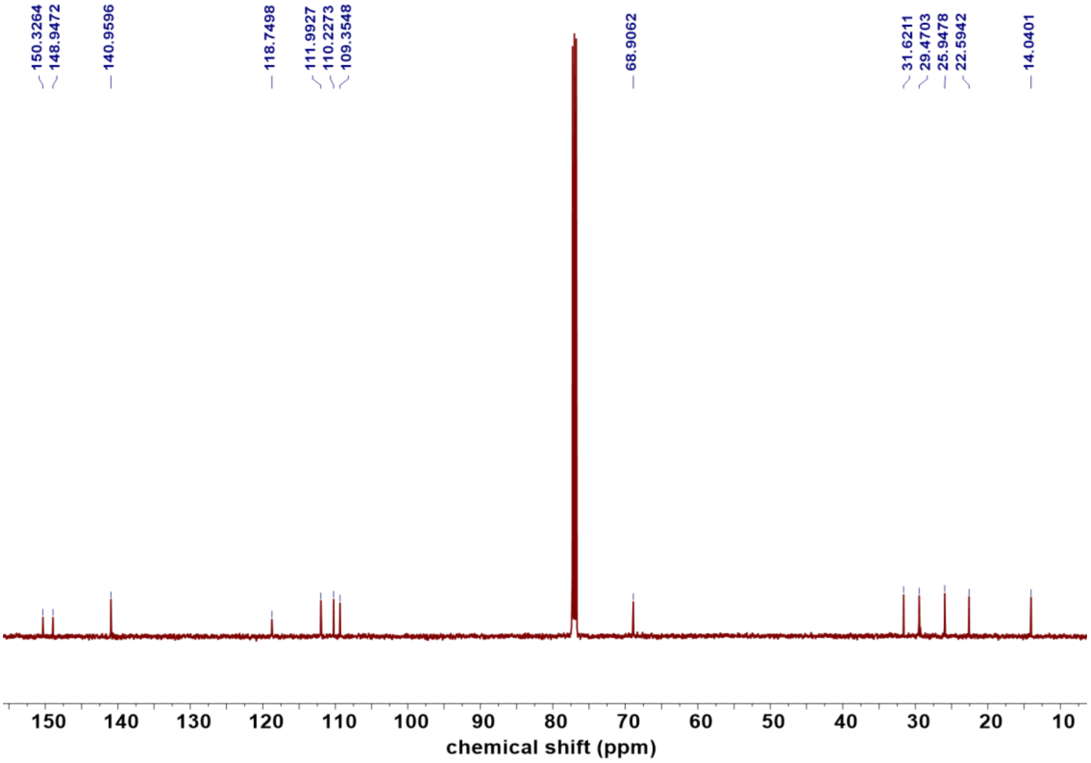

Supplementary Figure S25. ^{13}C NMR of **PhM-H**(O···O)

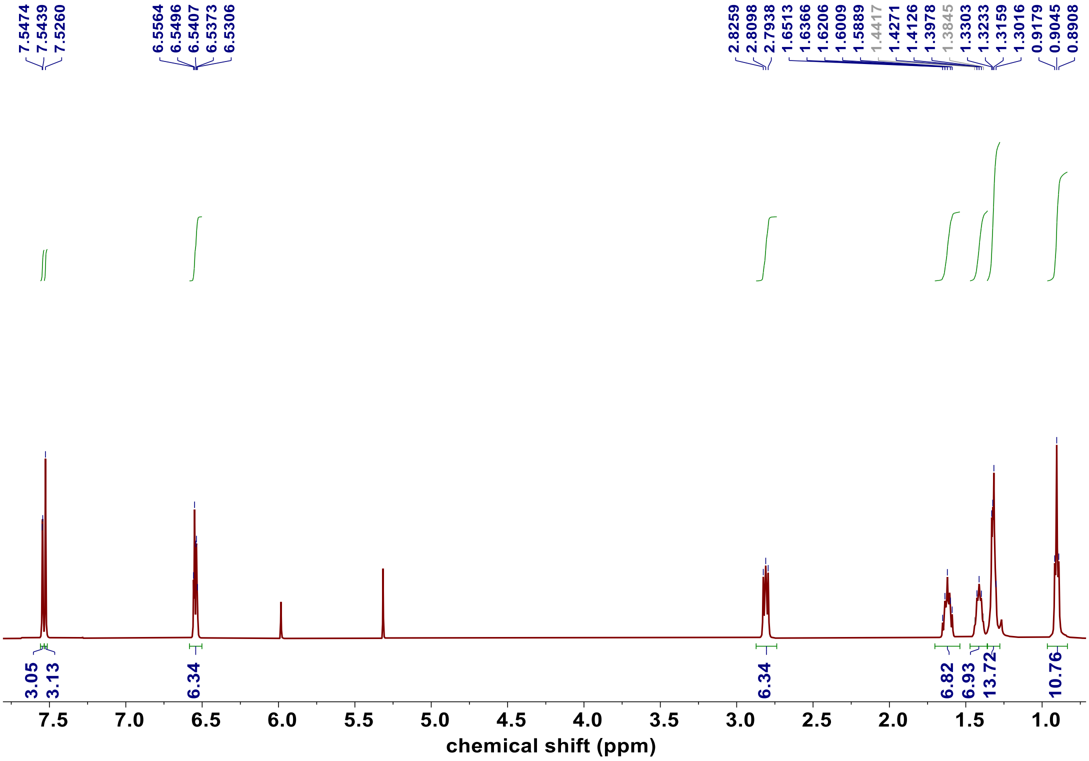

Supplementary Figure S26. ^{1}H NMR of **PhM-H**(O···C)

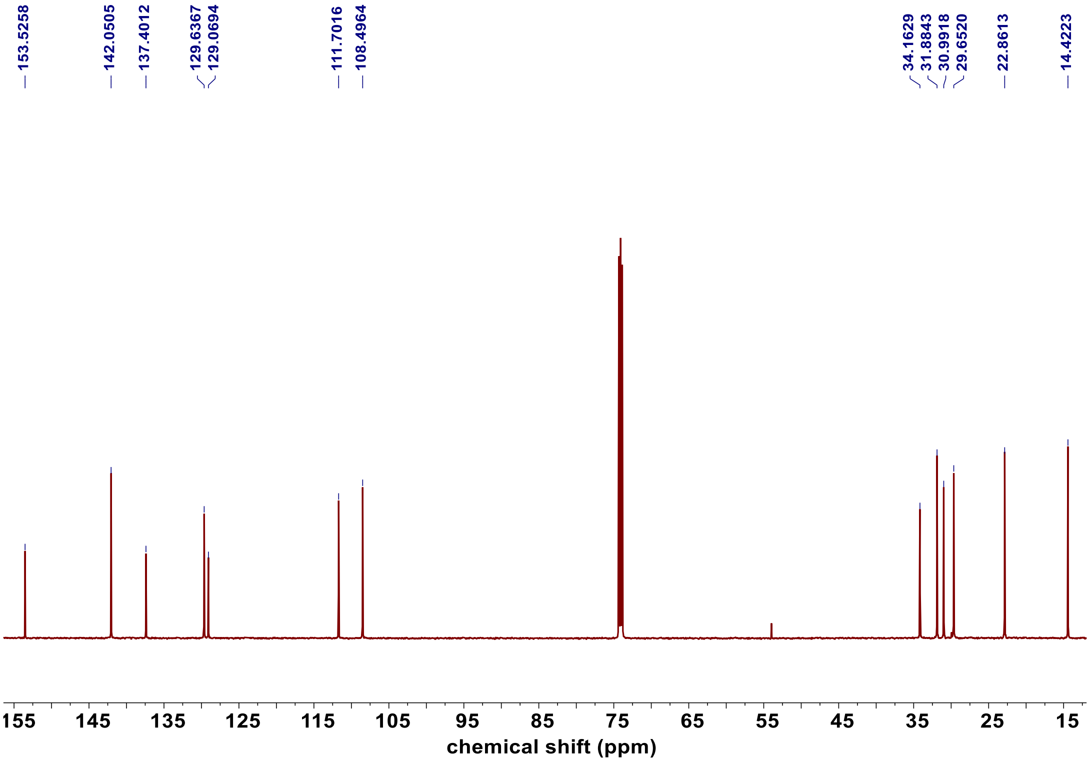

Supplementary Figure S27. ^{13}C NMR of **PhM-H**(O···C)

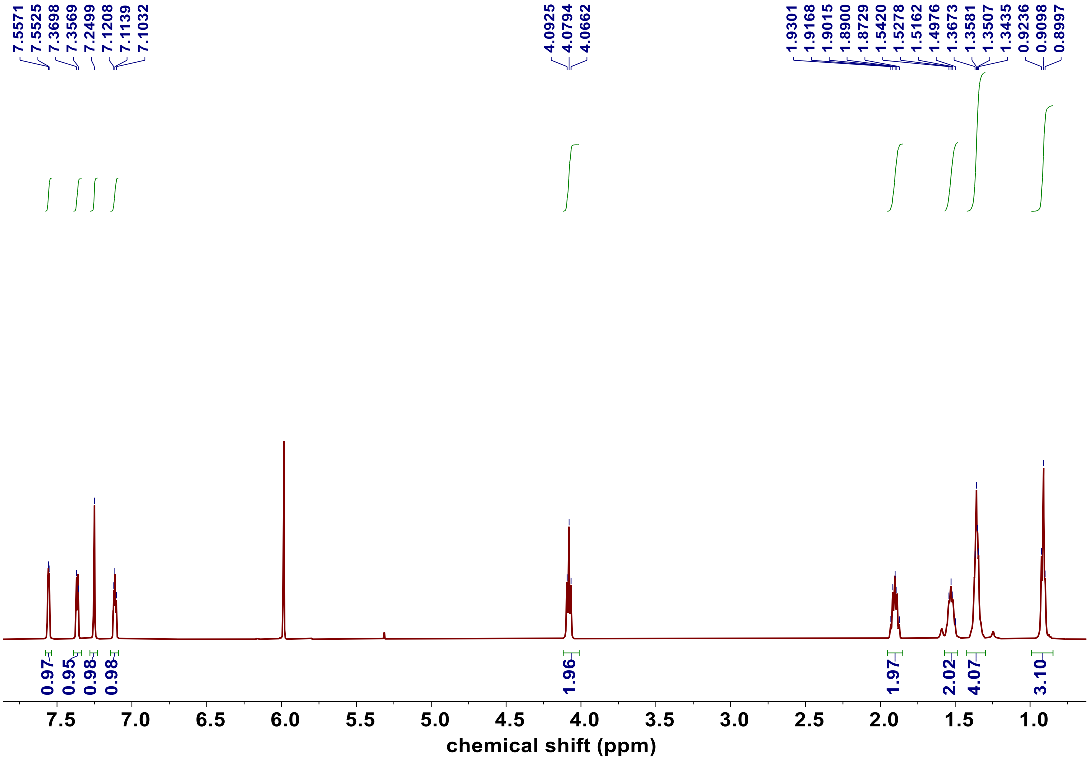

Supplementary Figure S28. ^{1}H NMR of **PhM**(S···O)

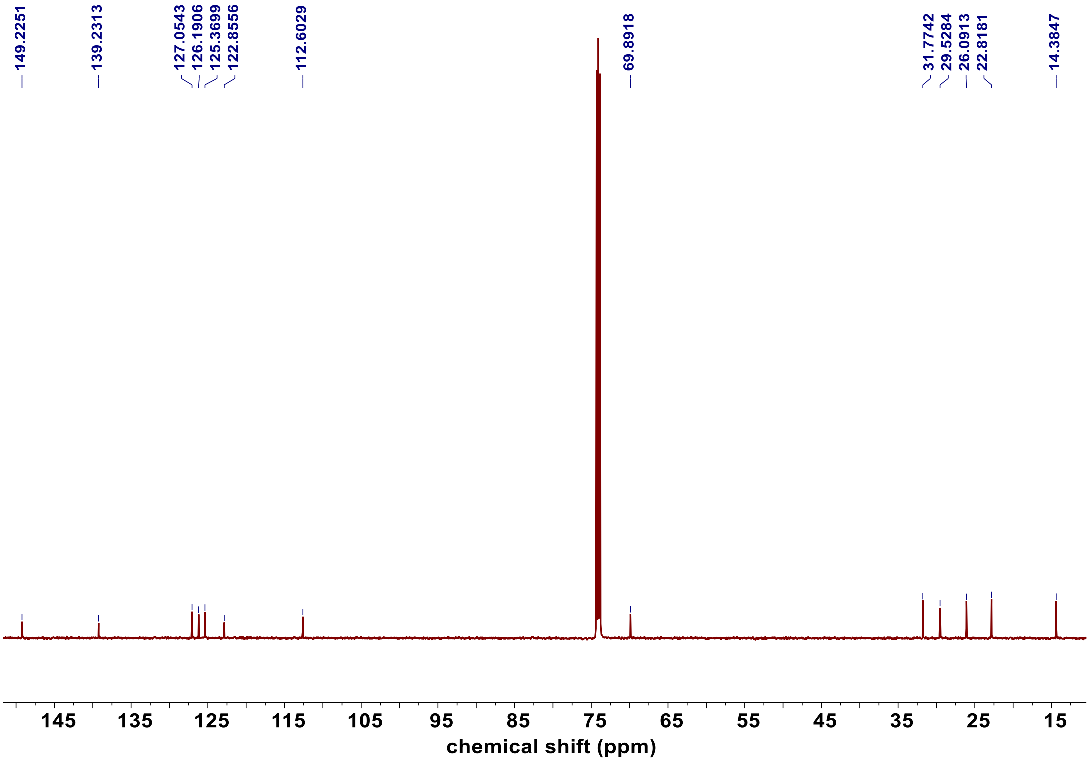

Supplementary Figure S29. ^{13}C NMR of **PhM**(S···O)

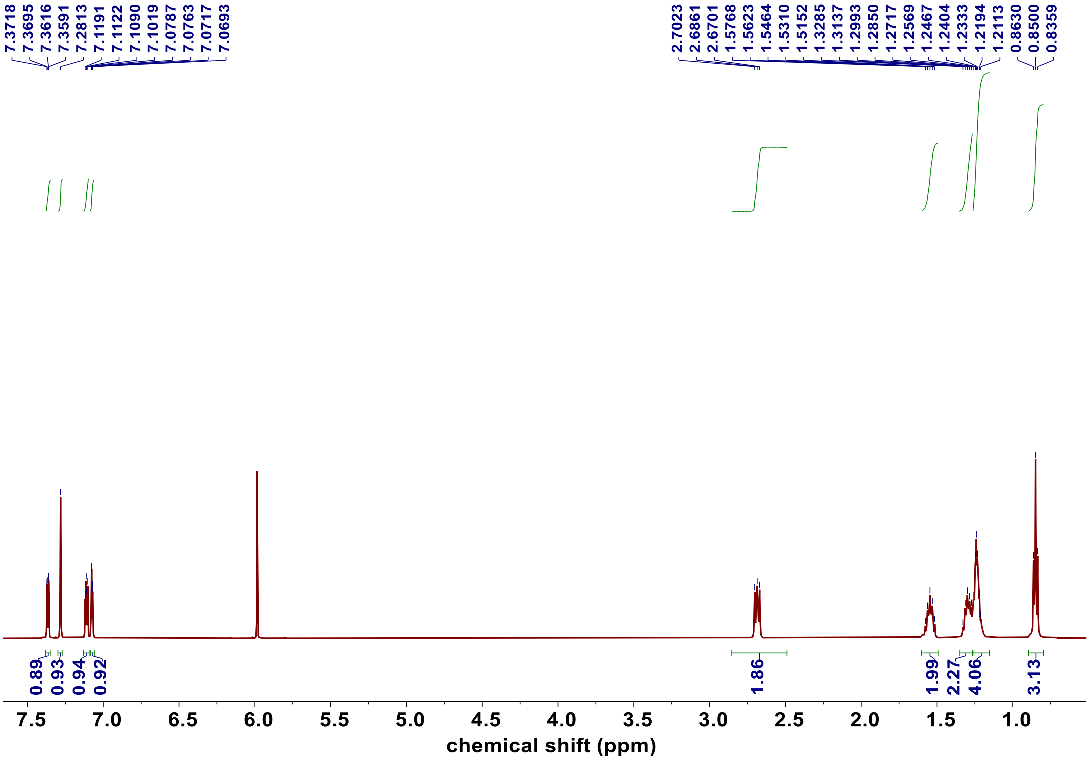

Supplementary Figure S30. ^{1}H NMR of **PhM**(S···C)

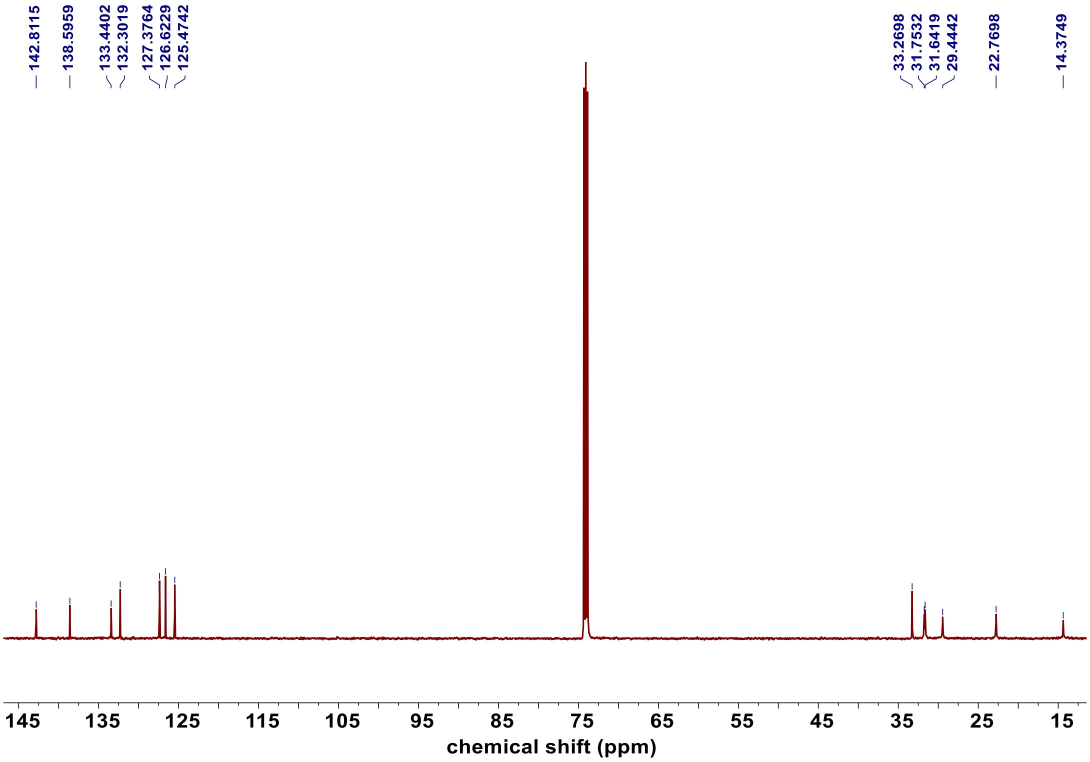

Supplementary Figure S31. ^{13}C NMR of **PhM**(S···C)

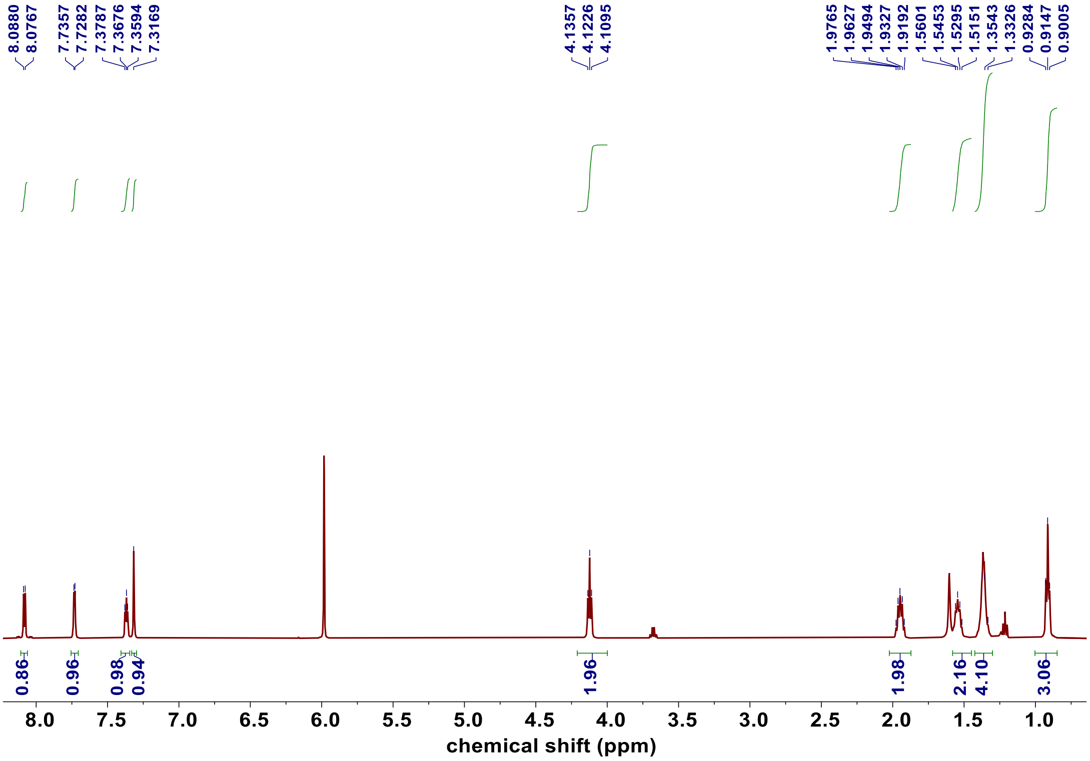

Supplementary Figure S32. ^{1}H NMR of **PhM**(Se···O)

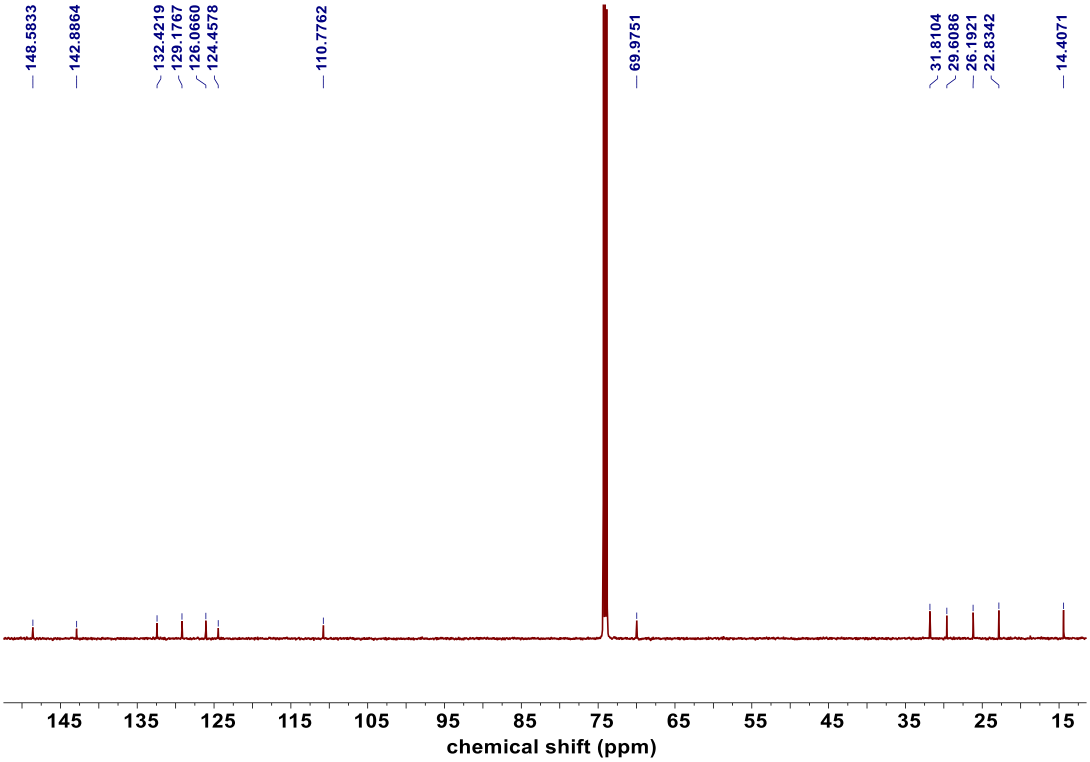

Supplementary Figure S33. ^{13}C NMR of **PhM**(Se···O)

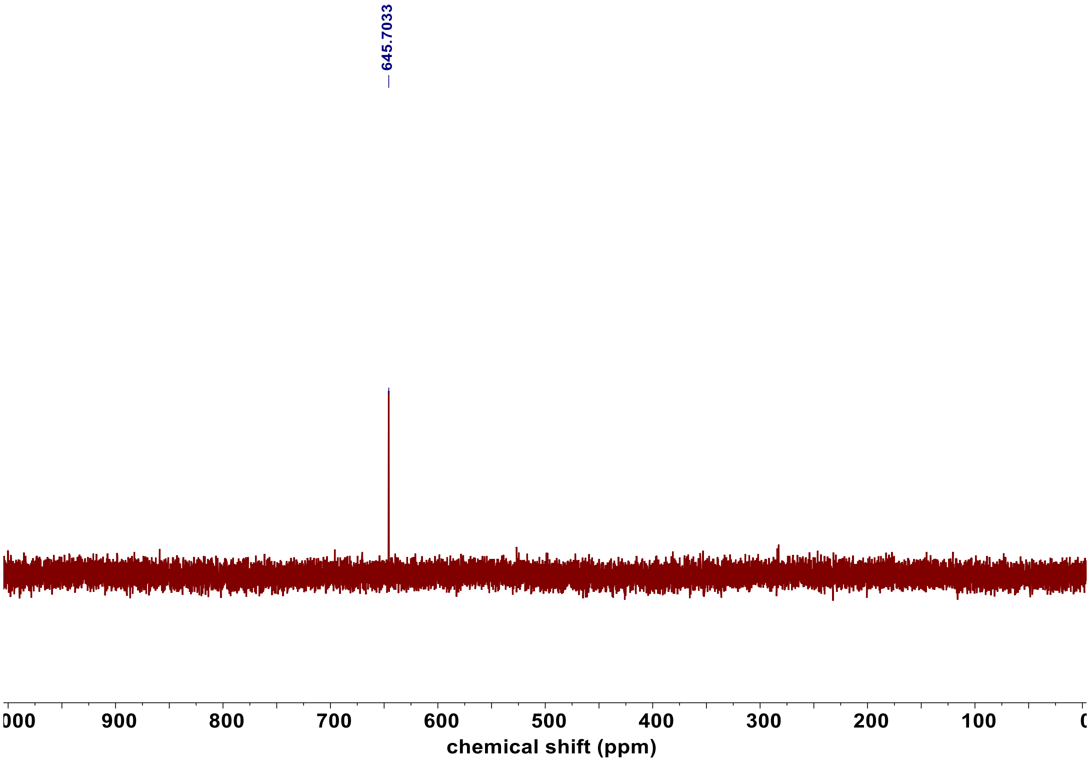

Supplementary Figure S34. ^{77}Se NMR of **PhM**(Se···O)

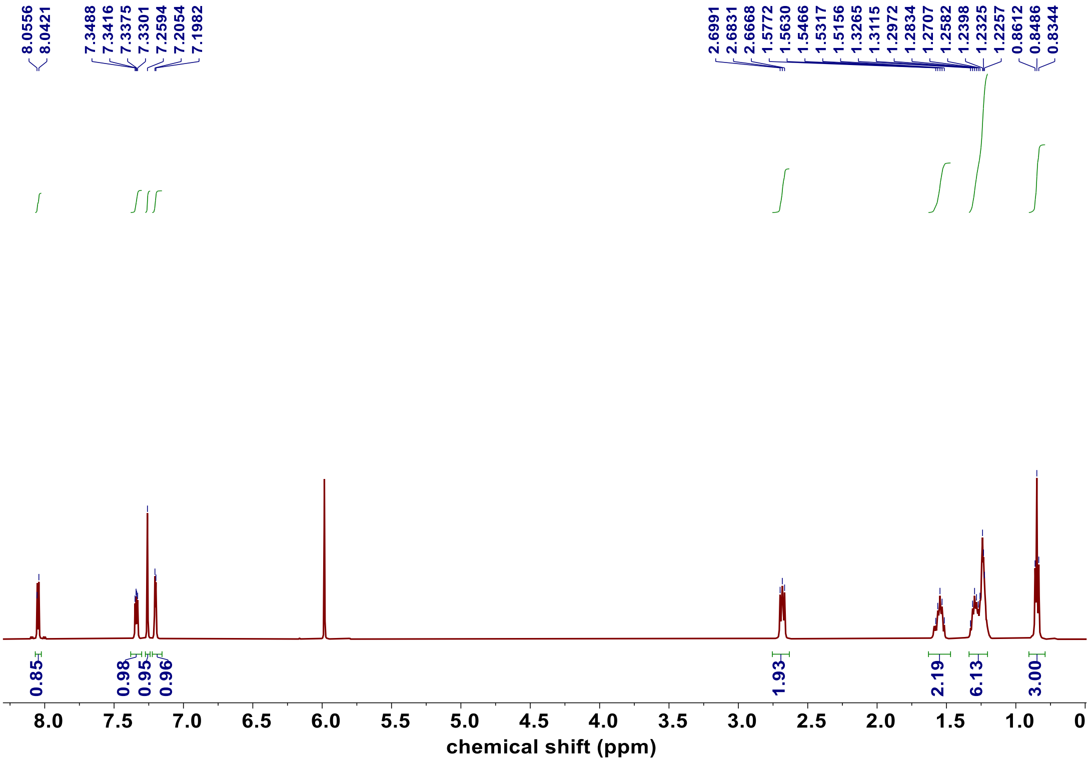

Supplementary Figure S35. ^{1}H NMR of **PhM**(Se···C)

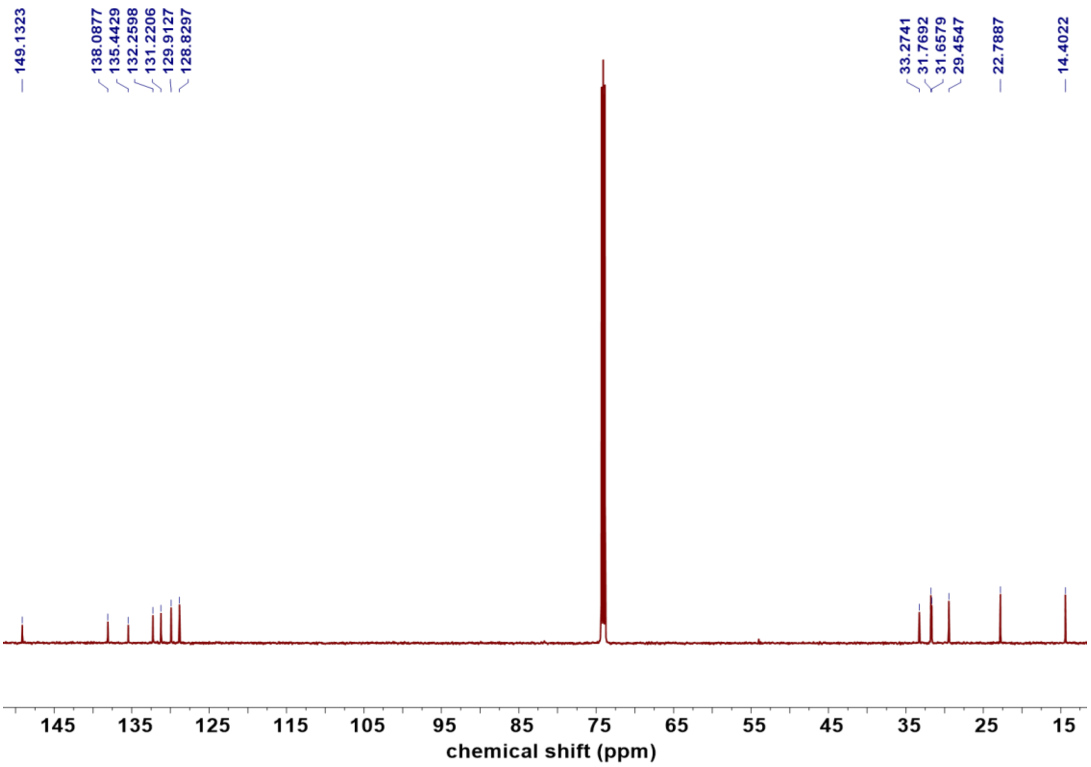

Supplementary Figure S36. ^{13}C NMR of **PhM**(Se···C)

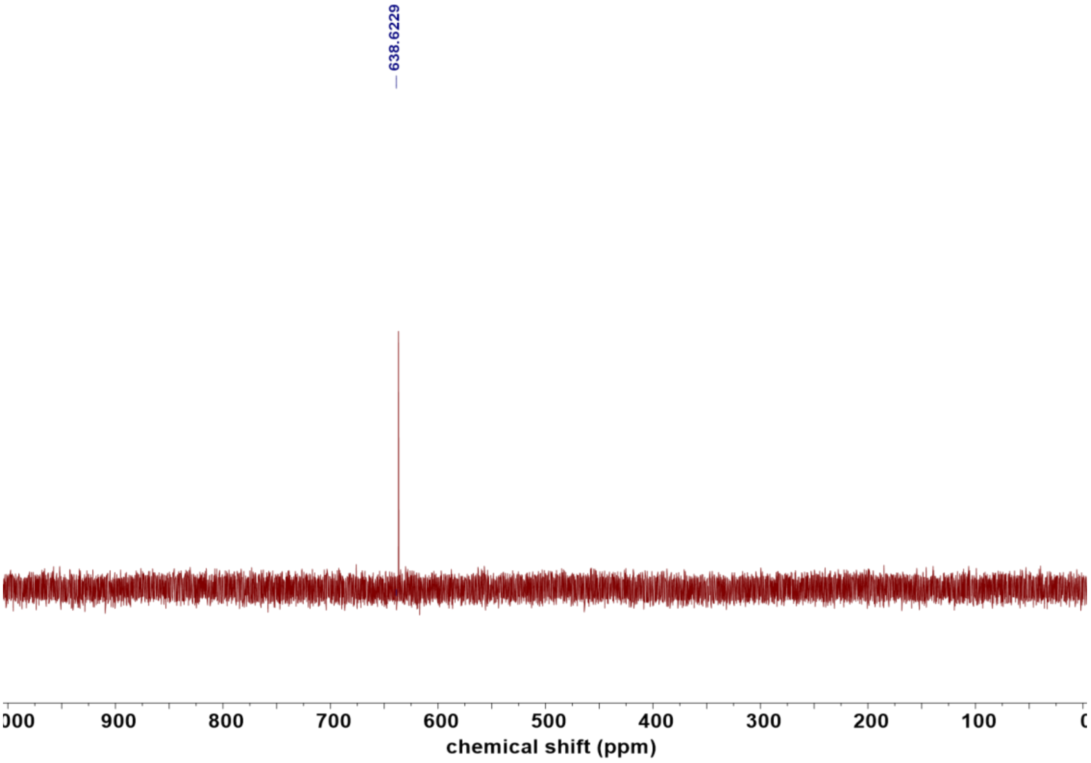

Supplementary Figure S37. ^{77}Se NMR of **PhM**(Se···C)

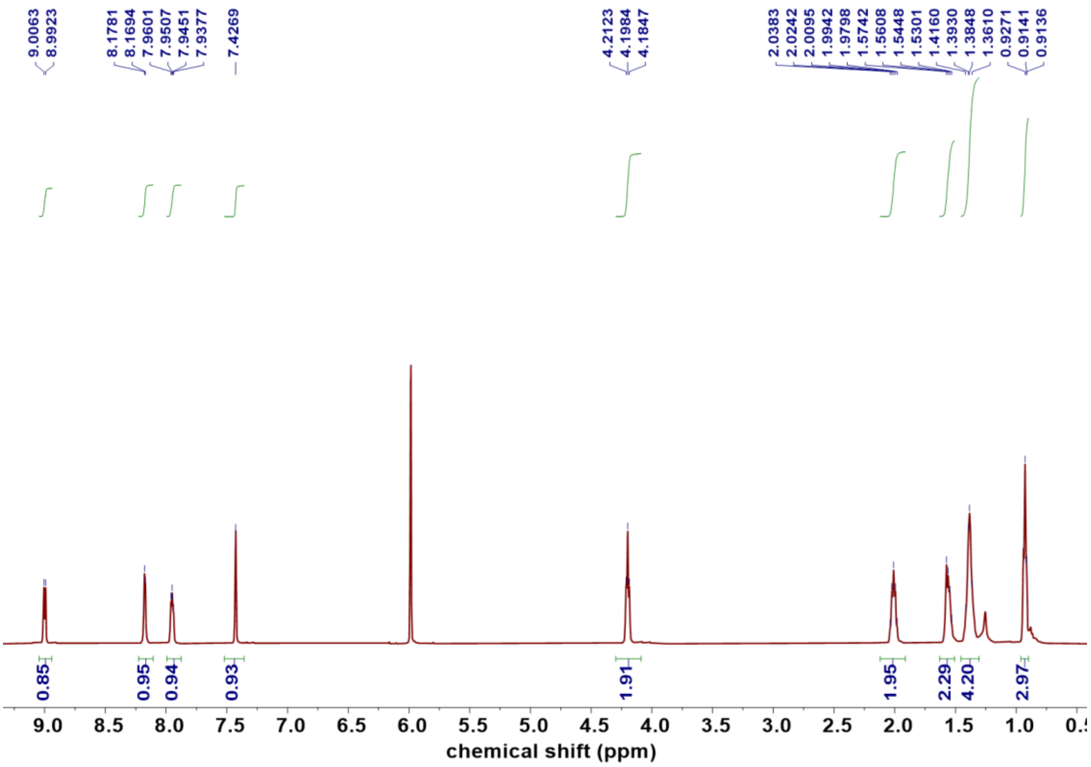

Supplementary Figure S38. ^{1}H NMR of **PhM**(Te···O)

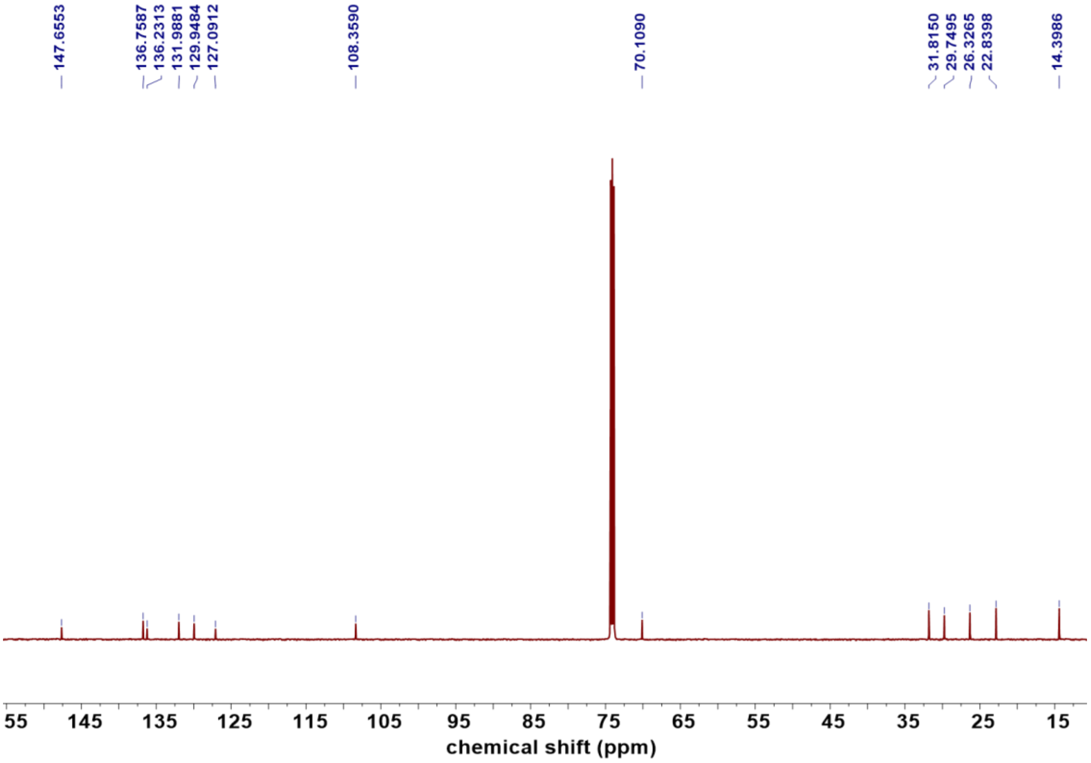

Supplementary Figure S39. ^{13}C NMR of **PhM**(Te···O)

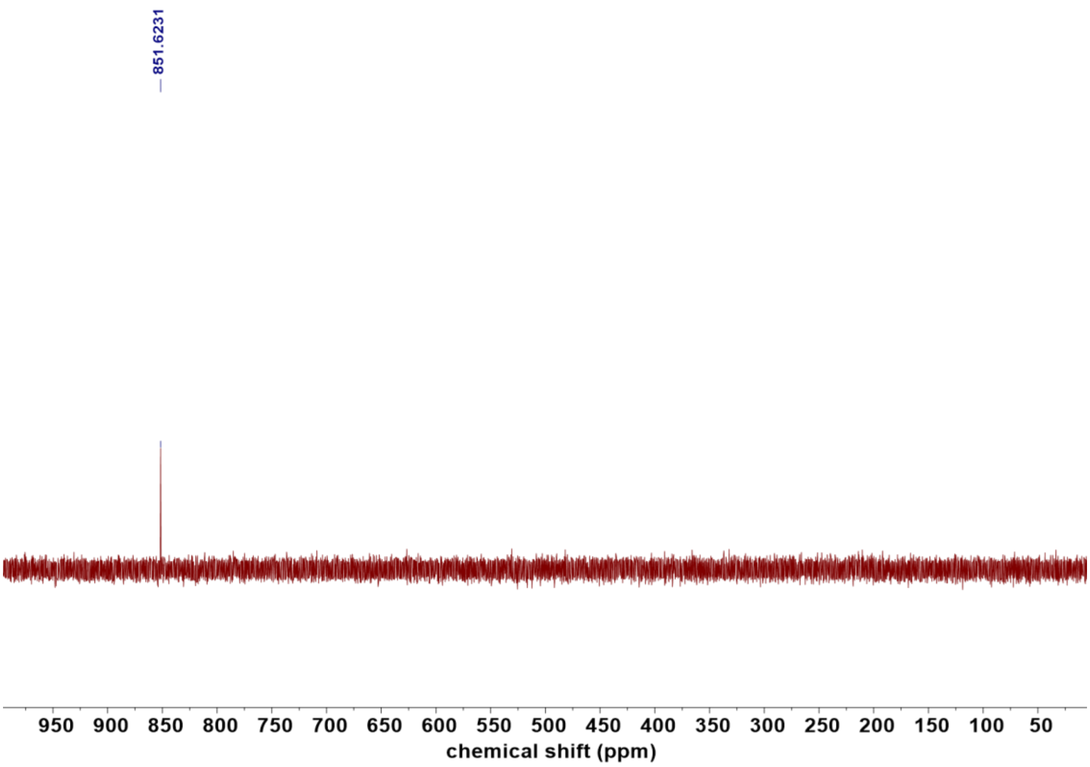

Supplementary Figure S40. ^{125}Te NMR of **PhM**(Te···O)

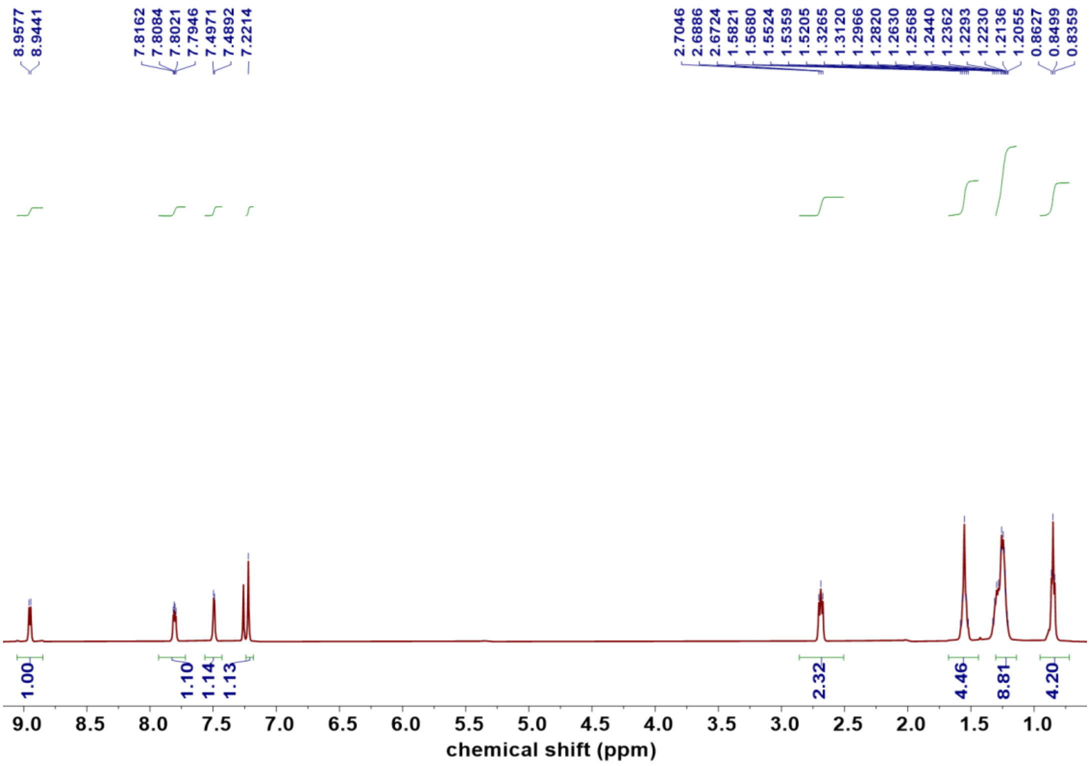

Supplementary Figure S41. ^{1}H NMR of **PhM**(Te···C)

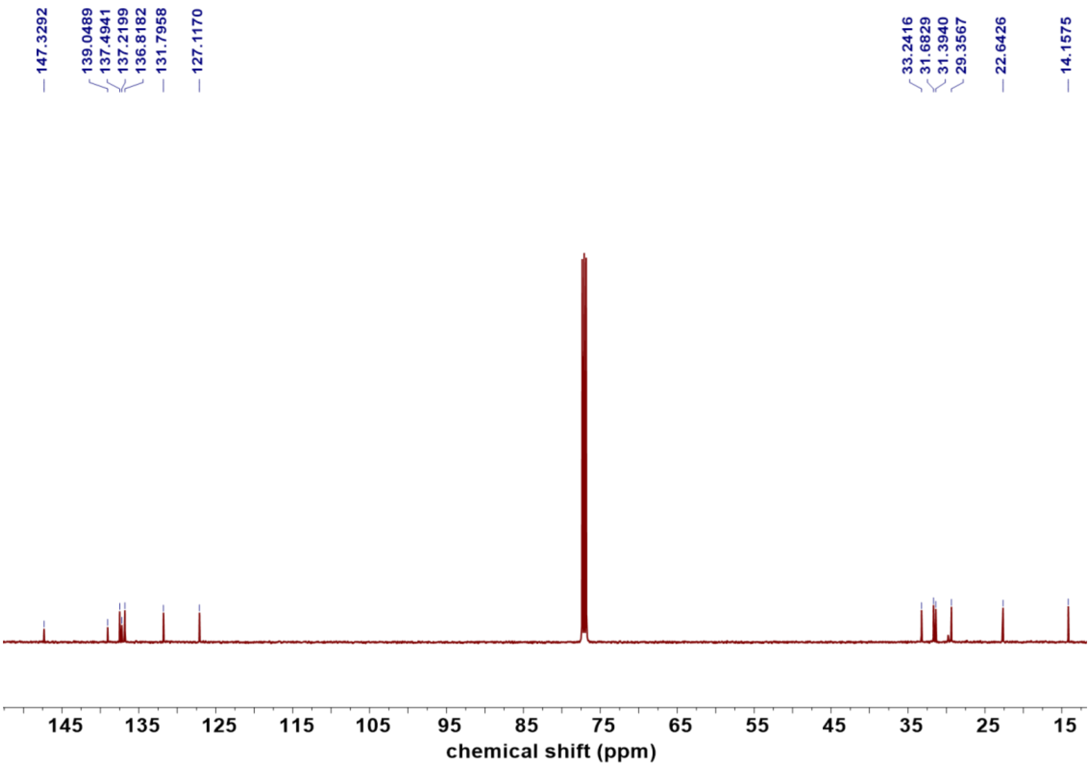

Supplementary Figure S42. ^{13}C NMR of **PhM**(Te···C)

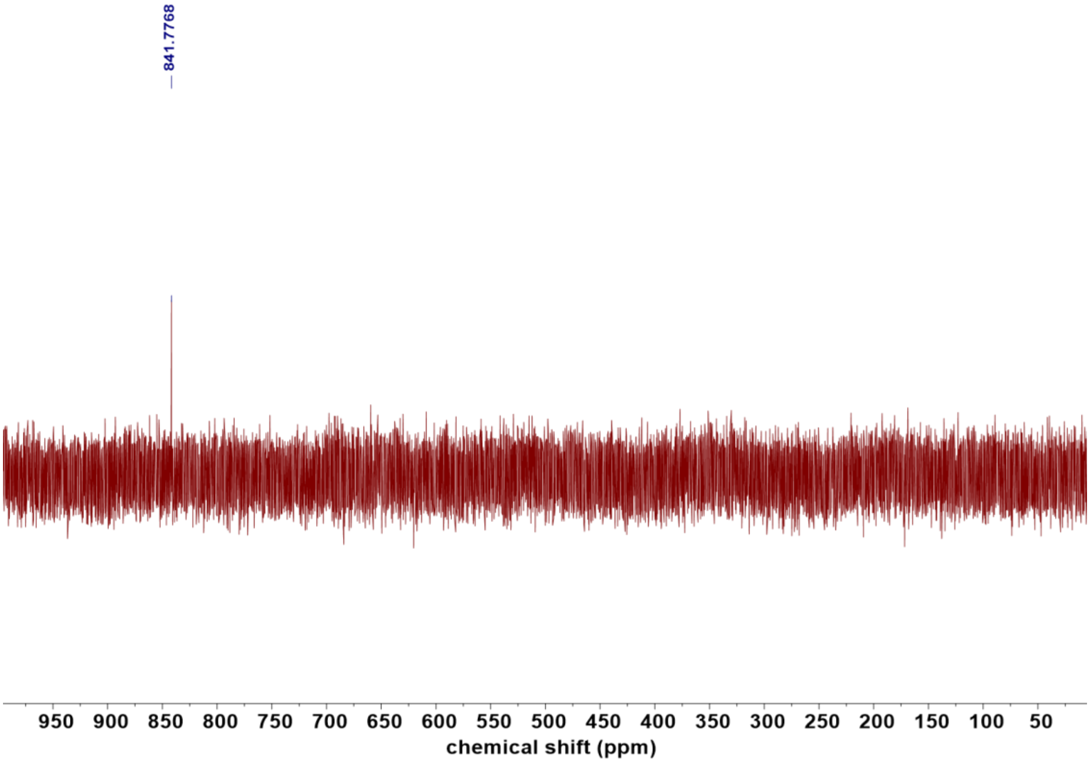

Supplementary Figure S43. ^{125}Te NMR of **PhM**(Te···C)

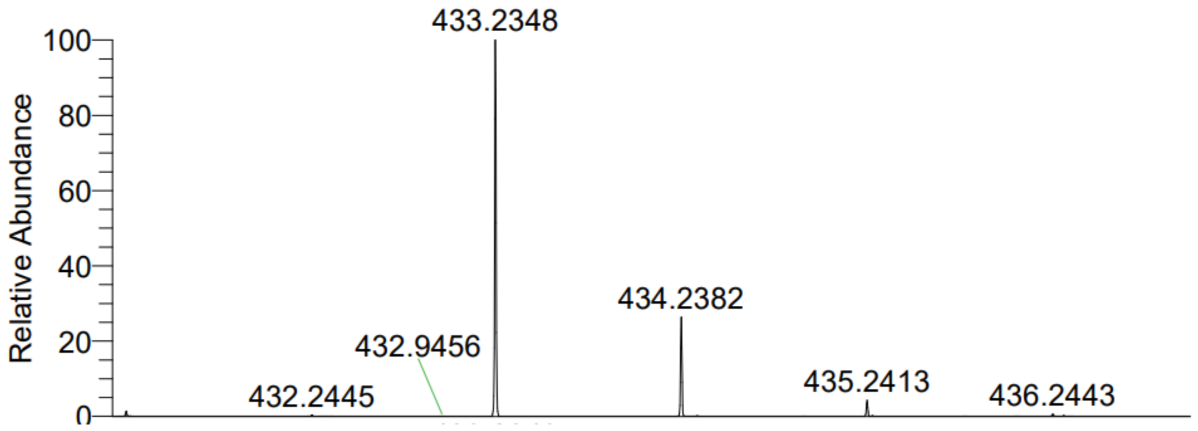

Supplementary Figure S44. LC-MS of **PhM**(O···O)

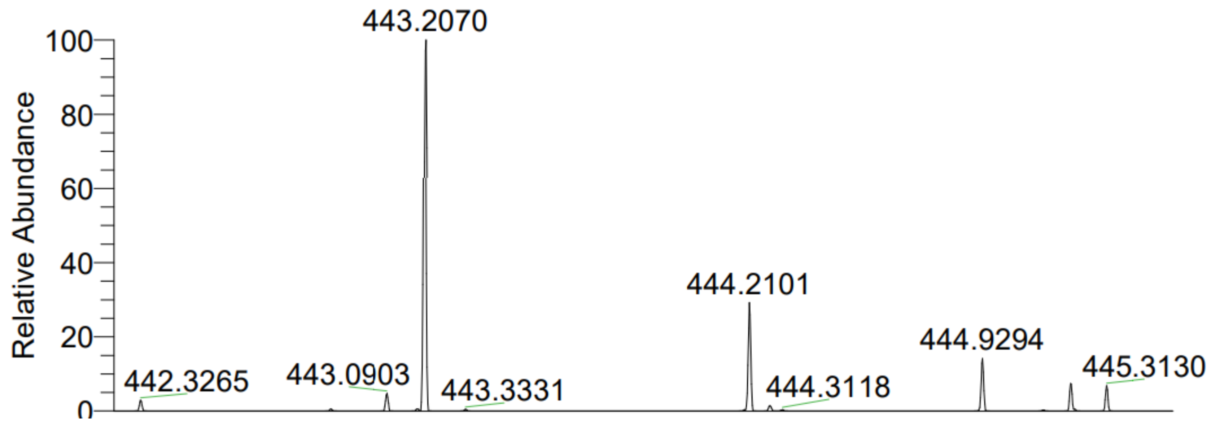

Supplementary Figure S45. LC-MS of **PhM**(S···O)

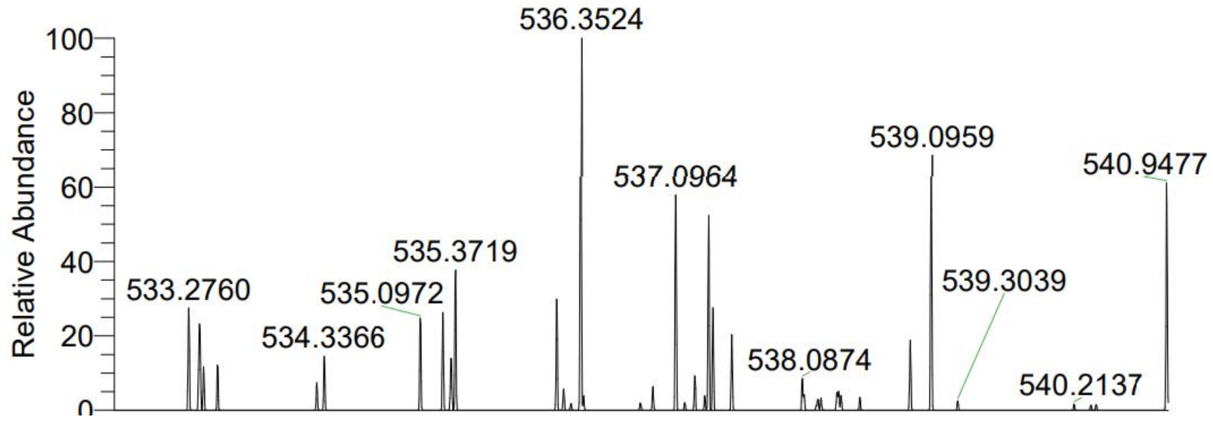

Supplementary Figure S46. LC-MS of **PhM**(Se···O)

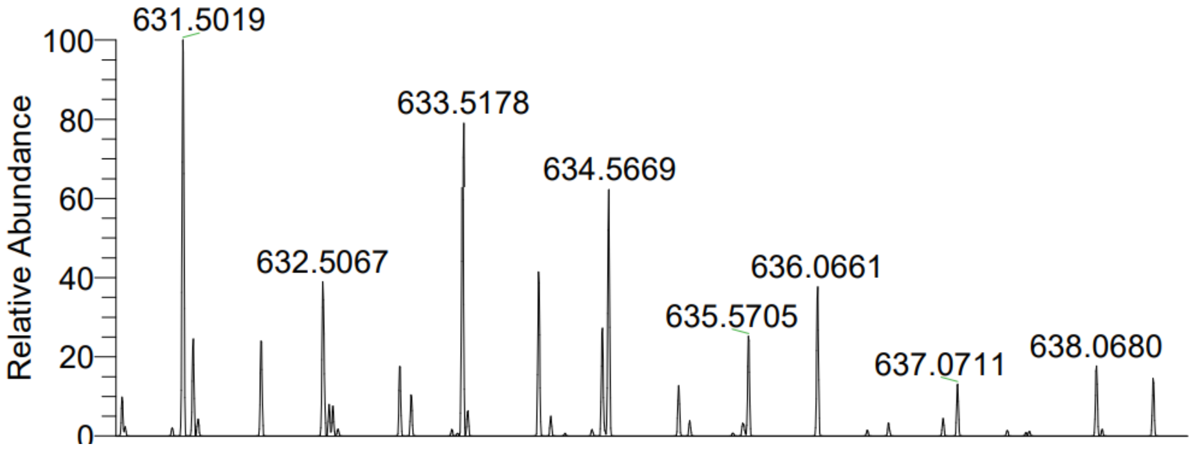

Supplementary Figure S47. LC-MS of **PhM**(Te···O)

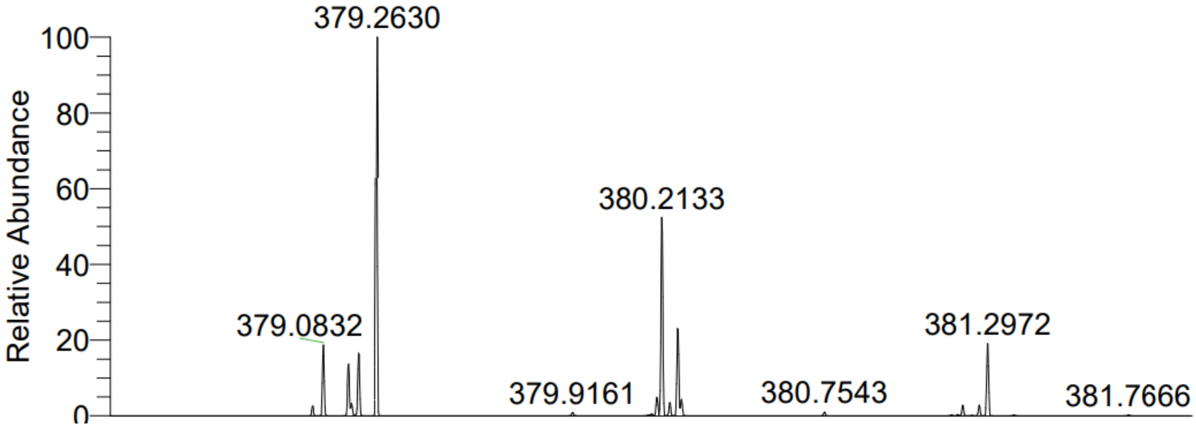

Supplementary Figure S48. LC-MS of **PhM**(O···C)

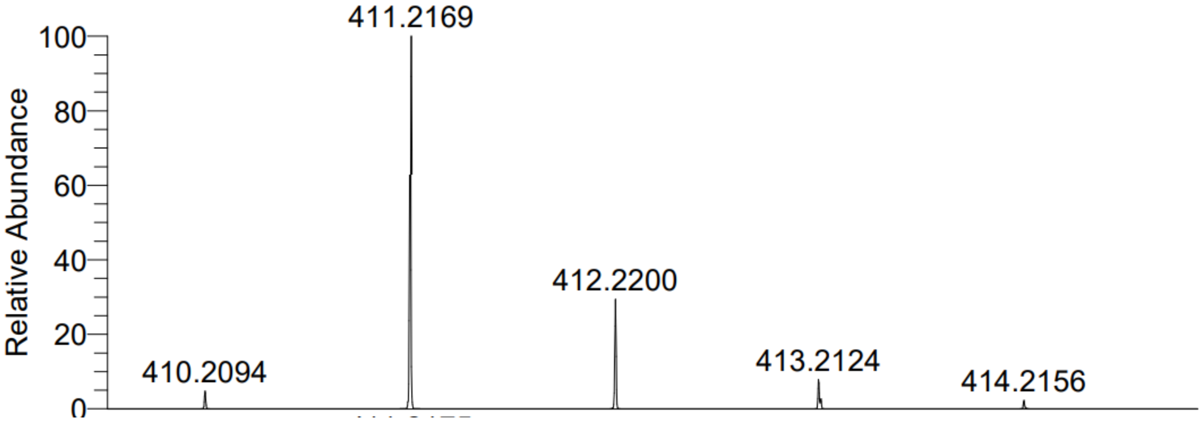

Supplementary Figure S49. LC-MS of **PhM**(S···C)

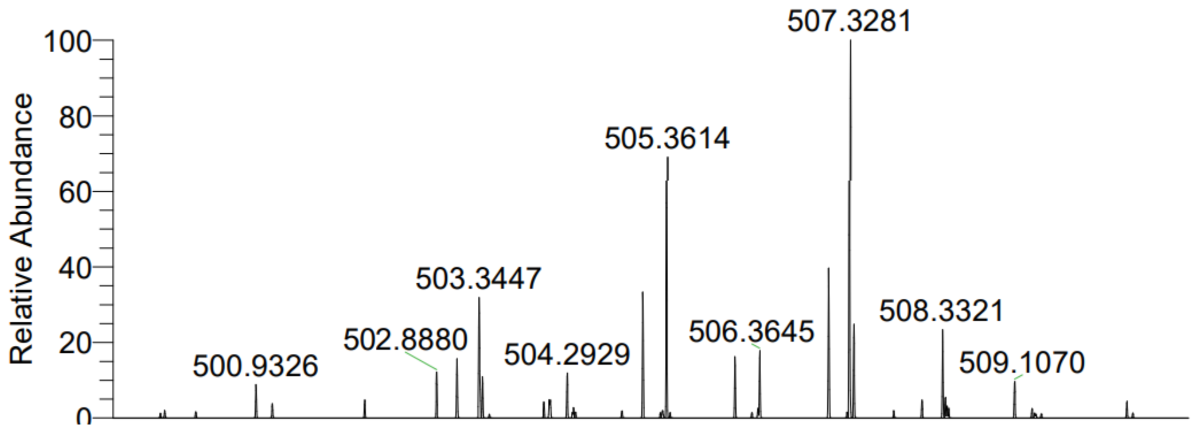

Supplementary Figure S50. LC-MS of **PhM**(Se···C)

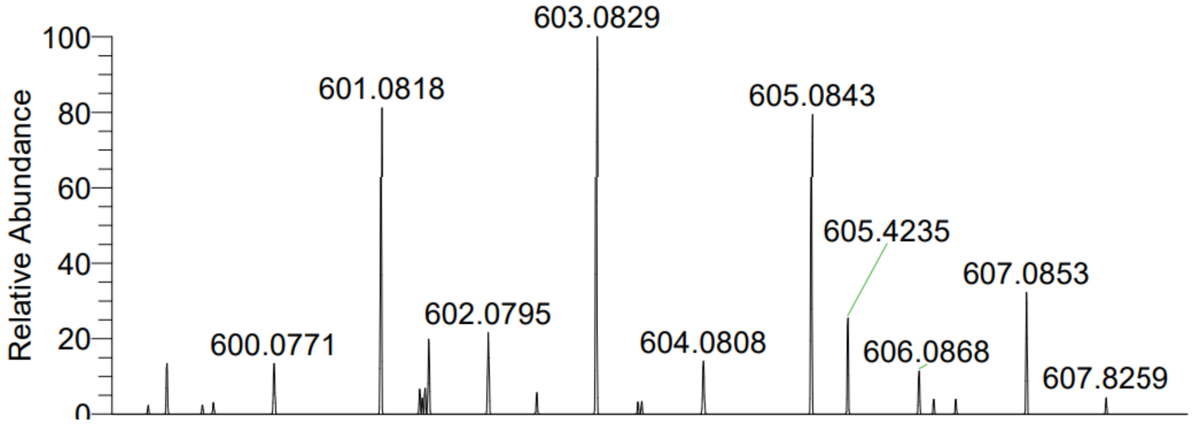

Supplementary Figure S51. LC-MS of **PhM**(Te···C)

14. Supplementary references

1. Huang, H. *et al.* Combining Electron-neutral Building Blocks with Intramolecular "Conformational Locks" Affords Stable, High-Mobility p- and n-Channel Polymer Semiconductors. *J. Am. Chem. Soc.* **134**, 10966-10973 (2012).
2. Hergue, N. *et al.* Facile Synthesis of 3-Alkoxy-4-cyanothiophenes As New Building Blocks for Donor-Acceptor Conjugated Systems. *Org. Lett.* **13**, 1762-1765 (2011).
3. Raimundo, J. M. *et al.* Push-Pull Chromophores based on 2,2 '-Bi(3,4-ethylenedioxythiophene) (BEDOT) π-Conjugatiug Spacer. *Tetrahedron. Lett.* **42**, 1507-1510 (2001).

4. Turbiez, M. *et al.* Design of Organic Semiconductors: Tuning the Electronic Properties of π-Conjugated Oligothiophenes with the 3,4-Ethylenedioxythiophene (EDOT) Building Block. *Chem. Eur. J.* **11**, 3742-3752 (2005).
5. Pomerantz, M., Amarasekara, A. S. & Dias, H. V. R. Synthesis and Solid-State Structures of Dimethyl 2,2 '-Bithiophenedicarboxylates. *J. Org. Chem.* **67**, 6931-6937 (2002).
6. Chaloner, P. A., Gunatunga, S. R. & Hitchcock, P. B. Redetermination of 2,2'-Bithiophene. *Acta Crystallographica Section C-Structural Chemistry.* **50**, 1941-1942 (1994).
7. Welch, G. C., Bakus, R. C., 2nd, Teat, S. J. & Bazan, G. C. Impact of Regiochemistry and Isoelectronic Bridgehead Substitution on the Molecular Shape and Bulk Organization of Narrow Bandgap Chromophores. *J. Am. Chem. Soc.* **135**, 2298-2305 (2013).
8. Hergue, N. *et al.* Evidence for the Contribution of Sulfur-Bromine Intramolecular Interactions to the Self-Rigidification of Thiophene-Based π-Conjugated Systems. *New J. Chem.* **32**, 932-936 (2008).
9. Sakamoto, Y., Komatsu, S. & Suzuki, T. Tetradecafluorosexithiophene: the First Perfluorinated Oligothiophene. *J. Am. Chem. Soc.* **123**, 4643-4644 (2001).
10. Kharandiuk, T. *et al.* Noncovalent Close Contacts in Fluorinated Thiophene–Phenylene–Thiophene Conjugated Units: Understanding the Nature and Dominance of O···H versus S···F and O···F Interactions with Respect to the Control of Polymer Conformation. *Chem. Mater.* **31**, 7070-7079 (2019).
11. Reed, A. E., Curtiss, L. A. & Weinhold, F. Intermolecular Interactions From a Natural Bond Orbital, Donor-Acceptor Viewpoint. *Chem. Rev.* **88**, 899-926 (1988).
