# Supplementary material for: A molecular descriptor of intramolecular noncovalent interaction for regulating optoelectronic properties of organic semiconductors
Source: Nat Commun. 2023 May 1;14:2500. doi: 10.1038/s41467-023-38078-4 (PMC10151346; doi:10.1038/s41467-023-38078-4)
Supplement: Supplementary file 3 — Description of Additional Supplementary Files [file 41467_2023_38078_MOESM3_ESM.pdf]

Description of Additional Supplementary Files

File name: Supplementary Data 1

Description: The single-crystal data of **PhM**(O···O) are summarized in Supplementary Data 1, and archived at the Cambridge Crystallographic Data Centre under the reference number CCDC-2152134.

File name: Supplementary Data 2

Description: The single-crystal data of **PhM**(Se···O) are summarized in Supplementary Data 2, and archived at the Cambridge Crystallographic Data Centre under the reference number CCDC-2152135.

File name: Supplementary Data 3

Description: The single-crystal data of **PhM**(Se···C) are summarized in Supplementary Data 3, and archived at the Cambridge Crystallographic Data Centre under the reference number CCDC-2152136.

File name: Supplementary Data 4

Description: The single-crystal data of **PhM**(S···O) are summarized in Supplementary Data 4, and archived at the Cambridge Crystallographic Data Centre under the reference number CCDC-2152139.

File name: Supplementary Data 5

Description: The single-crystal data of **PhM**(S···C) are summarized in Supplementary Data 5, and archived at the Cambridge Crystallographic Data Centre under the reference number CCDC-2152140.

File name: Supplementary Data 6

Description: The single-crystal data of **PhM**(Te···O) are summarized in Supplementary Data 6, and archived at the Cambridge Crystallographic Data Centre under the reference number CCDC-2152141.

File name: Supplementary Data 7

Description: Atomic coordinates of the optimized geometries for the studied systems.
